# Mutual, spatially limited control of meiotic DNA break formation by Mre11–Rad50–Nbs1 DNA repair complex and Tel1 (ATM) protein kinase

**DOI:** 10.1093/nar/gkaf1405

**Published:** 2025-12-22

**Authors:** Randy W Hyppa, Gerald R Smith

**Affiliations:** Fred Hutchinson Cancer Center, Division of Basic Sciences, Seattle, WA 98109-1024, United States; Fred Hutchinson Cancer Center, Division of Basic Sciences, Seattle, WA 98109-1024, United States

## Abstract

Meiotic recombination is initiated by DNA double-strand breaks (DSBs); factors that control DSB frequencies are important to produce viable progeny. In many organisms, the ATM (Tel1) protein kinase prevents excessive meiotic DSBs, especially nearby DSBs on the same chromatid. Normally, two close DSBs are less frequent than expected from independence, a feature called DSB interference, which is lost in *tel1Δ* mutants. In the fission yeast *Schizosaccharomyces pombe*, high-level DSB formation depends on linear elements, Hop1, and meiotic cohesin complexes; we show here that these complexes impart competition between nearby DSB sites. When these complexes are impaired, Tel1 substantially represses DSB formation, and in its absence, two close DSBs on the same chromatid occur frequently and manifest high negative interference. After mitotic DNA damage, the conserved Mre11–Rad50–Nbs1 (MRN) complex is required for DNA resection, and the Tel1 kinase activity is needed to complete DSB repair. We found that during meiosis *mre11Δ* and *rad50Δ* mutants, like *tel1Δ* mutants, lack DSB interference and display highly negative DSB interference in meiotic complex mutants. Thus, MRN at a DSB site appears critical for Tel1 function in meiosis and reveals a complex interplay of positive and negative factors controlling meiotic DSB formation.

## Graphical Abstract

**Graphical Abstract:**
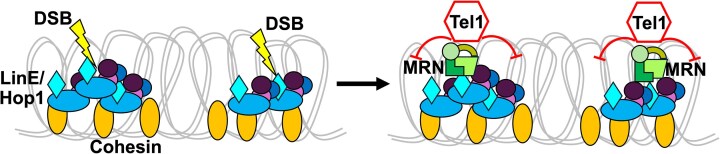

## Introduction

Meiosis produces haploid gametes from diploid progenitor cells by following DNA replication with two successive cell divisions. Preceding the first cell division is meiotic recombination, in which programmed DNA double-strand breaks (DSBs) are created as an initiation point for the engagement of homologous chromosomes necessary for an effective meiosis. The repair of the DSBs has two beneficial consequences: reciprocal genetic rearrangements, called crossovers, create genetically diverse progeny, and the tension created by crossovers aids the proper segregation of chromosomes [1]. The mechanisms that regulate DSB formation have been studied in many organisms but are not fully understood [2]. Here, we examine this regulation in a simple eukaryote, the fission yeast *Schizosaccharomyces pombe*.

The protein complexes involved in breaking meiotic DNA are highly conserved and well characterized [3], the most prominent being Spo11 (called Rec12 in *S. pombe*), which contains the active site for DSB formation [4–9]. After replication, meiotic cohesin complexes, containing the meiosis-specific subunits Rec8 and Rec11 in *S. pombe*, are loaded onto sister chromatids and hold them together [10–16]. Cells lacking cohesin have reduced but substantial DSBs, reduced crossovers, and aberrant segregation, leading to poor viability [11, 13, 17–20]. In *S. pombe*, an important early event is the phosphorylation of Rec11 by the casein kinases Hhp1–Hhp2, which is essential for the interaction of Rec11 with the linear element (LinE) component Rec10; DSBs formed by Rec12 require this interaction [21, 22]. Rec10 also binds to and colocalizes with the meiotic HORMAD protein Hop1 [23, 24], which is required for high-level recombination and DSB formation in most organisms studied [25]. Artificial targeting of Hop1 promotes DSB formation in normally “cold” DSB regions of *S. cerevisiae* [26].

The LinEs, composed of Rec25, Rec27, Mug20, and Rec10, localize to stretches of the meiotic chromosomes but do not run the entire length [23, 27, 28, 29]. They are functionally related to the synaptonemal complex (SC) proteins of many eukaryotes. The SC is a tripartite structure in which a set of proteins comprising the axial element (AE) localizes along the chromosome axis of each pair of sisters (homologs). The AEs are connected by the central elements (CE), creating a connection between the two homologs [30]. The components of the SC are well conserved, though the timing of SC formation varies among species. *S. pombe* does not have a complete SC; rather, the LinEs are functionally equivalent to AEs [23], and they share similarity in protein structure with the AE coiled-coil proteins of other species [31, 32]. LinE formation is cohesin-dependent in *S. pombe*, like chromosome axis formation in many species examined [10, 11, 12, 23, 33–36]. Cohesin and LinE deletion mutants in *S. pombe* have similar meiotic phenotypes: reduced DSBs and a loss of crossovers in a region-dependent manner, the exception being a *rec10* null mutant, which is nearly as DSB- and recombination-deficient genome-wide as a *rec12* null mutant [18, 28, 29, 37].

After a DSB is made, the highly conserved Mre11–Rad50–Nbs1 (MRN) complex initiates DSB repair via the nuclease activity of Mre11 [38, 39]. Coupled with the necessary accessory protein Ctp1, Mre11 clips one DNA strand and removes short ssDNA oligonucleotides covalently linked at their 5′ end to Spo11 (or Rec12 in *S. pombe*) [40–42]. This creates a 3′ ssDNA end that can be lengthened by further resection of the 5′ end by MRN and Exo1 exonuclease [43–46]. Aided by the DNA strand-exchange proteins Rad51 and Dmc1, this long 3′-ended ssDNA invades an intact dsDNA molecule (homolog or sister) for repair by homologous recombination [47].

MRN mutants in different species have varying DSB phenotypes. Both Mre11 and Rad50, but not Nbs1, are required for meiotic DSB formation in the budding yeast *S. cerevisiae* and the nematode *Caenorhabditis elegans* [48–51]. In these species, Mre11 is present at the time of DSB formation at special chromosomal sites (hotspots) at which DSBs (and recombinants) arise at high frequency [52]. However, in *Coprinus cinereus, Arabidopsis thaliana, S. pombe, Tetrahymena thermophila*, and mice, meiotic DSBs are formed in MRN mutants [53–57], though at a slightly reduced level in *S. pombe*. In all MRN mutants tested where DSBs are made, they are not repaired due to the lack of DNA resection, there are few or no crossovers, and meiotic viability is poor [58]. DSBs can be repaired through an alternative pathway of non-homologous end joining (NHEJ), in which, instead of strand invasion of duplex DNA, two broken DNA ends are ligated together [59]. NHEJ is thought to be restricted during meiosis by the presence of Spo11 (Rec12) bound to the DSB, by MRN binding to DSB ends and promoting homologous recombination [60], and by phosphorylation of SC components [61].

The conserved protein kinases ATR and ATM are necessary for responding to mitotic DNA damage and activating a checkpoint by phosphorylating multiple proteins to halt the cell cycle and thus allow DNA repair. MRN plays a critical role in recruiting and activating ATM and ATR to sites of DNA damage [62]. In meiosis, both ATR and ATM have roles in responding to and regulating DSB formation [63–65], and in both budding yeast and mice, ATM (called Tel1 in yeasts) is needed for full-length DNA resection [46, 66]. Additionally, ATM (Tel1) restricts the number of DSBs during meiosis [65, 67–73]; in budding yeast total DSBs increase 2-fold and in mice 10-fold. ATM (Tel1) prevents the same chromatid from being broken multiple times at or near a given site, as frequent, closely spaced DNA double-cuts are seen in yeast and mice ATM mutants [67, 73–76]. In wild-type meiosis, the formation of one DSB interferes with the formation of another DSB nearby, a feature known as DSB interference. This feature requires Tel1 in both budding and fission yeasts [67, 73]. Budding yeast with mutations in Xrs2 (the Nbs1 homolog) lose DSB interference [77], and mice with a meiotic knockout of Mre11 have frequent, closely spaced (<1 kb) double-cut DNA during meiosis [56], evidence that MRN plays a role in ATM (Tel1) activation during meiosis. ATM and ATR phosphorylate Hop1 to stimulate inter-homolog DSB repair, but this phosphorylation is dispensable for DSB formation [78, 79].

We analyzed DSB formation in mutants of cohesin, LinEs, and Hop1 in *S. pombe*, with and without Tel1 present. We observed that DSBs were strongly reduced, and surprisingly, some, but not all, of the DSBs were restored when Tel1 was removed. Tel1 kinase activity reduced DSB formation when the cohesin or LinE complexes were aberrant or missing. Strong DSB hotspots compete with weaker ones [73, 80–84], and here we show DSB competition is dependent on both cohesin and LinEs. Additionally, the amount of double-cut DNA was very strongly increased in cohesin, LinE, or Hop1 mutants without Tel1, resulting in very strong negative DSB interference; that is, instead of a first DSB interfering with and preventing a second DSB, the presence of a DSB stimulated the formation of a second. We also show that *mre11Δ* and *rad50Δ*, but not *nbs1Δ* or certain point mutants of the MRN complex, have more double-cut chromatids, resulting in spatially limited negative DSB interference like *tel1Δ*. When *mre11Δ* or *rad50Δ* was combined with a cohesin mutant, the phenotype was again like that of *tel1Δ* – increased double-cut chromatids and strong negative DSB interference. This suggests that, as seen in mice [56], the MRN complex, but not MRN nuclease activity, is needed to activate Tel1 to restrict meiotic DSBs. We infer that MRN activates the kinase activity of Tel1 to block further DSB formation after a limited number of DSBs are formed in a LinE-bound cluster of hotspots. These results support our previous DSB hotspot clustering model for the control of meiotic DSB formation in *S. pombe* [73] (see the “Discussion” section).

## Materials and methods

### *Schizosaccharomyces pombe* growth materials and methods

Media for cell growth were yeast extract liquid (YEL) and modified Edinburgh minimal medium number 2 (EMM2*) or their agar-containing derivatives [85]. Sporulation, including mating, was on sporulation agar (SPA). Defined media were supplemented with all required nutrients at 100 µg/ml; for adenine-requiring strains, YEL was supplemented with adenine and uracil at 100 µg/ml. Methods were as in [85].

### DNA DSB analysis

**Figure 1.**
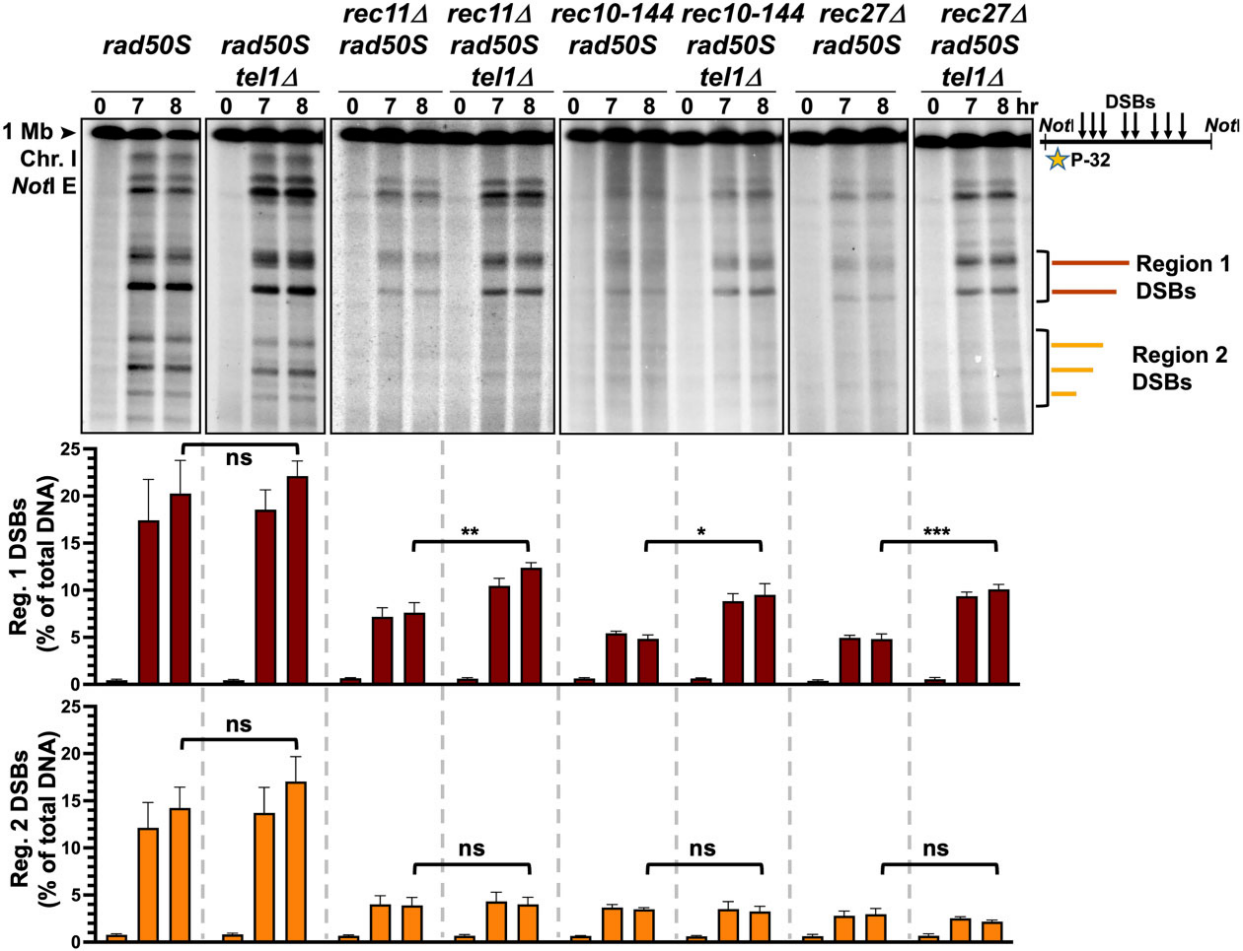
Region-specific meiotic DSBs are increased by *tel1Δ* in cohesin and linear element mutants. *Schizosaccharomyces pombe* strains with the *pat1-as* (L95G) mutation were induced into synchronous meiosis at 25°C. DNA was harvested from pre-meiotic cells at 0 h and in meiotically induced cells at 7 and 8 h, when DSB formation is complete and MI begins; *rad50S* strains were used to assay total (accumulated) DSBs. Agarose-embedded DNA was digested with NotI and separated by pulsed-field gel electrophoresis (PFGE). Images are of Southern blot hybridizations with [^32^P]-labelled DNA probes. DSBs were measured in cohesin mutant *rec11*Δ and LinE mutants *rec10-144* and *rec27*Δ across an ~0.5 Mb section of Chromosome I (designated Regions 1 and 2), in both *tel1^+^* and *tel1*Δ strains. DSB frequencies at 0, 7, and 8 h (as % of total DNA) are represented in the graphs beneath the blot images: upper graph, the sum of DSBs in Region 1; lower graph, the sum of DSBs in Region 2. For each strain, the mean and SEM are shown from three to six experiments. The [^32^P]-labelled DNA probe was located on the left end of the 1 Mb NotI E fragment, as indicated by the star in the scheme on the right. Two-tailed *P*-values calculated by unpaired *t*-test were ns (not significant, >.05), *(<.05), **(<.005), and ***(<.0005).

**Figure 2.**
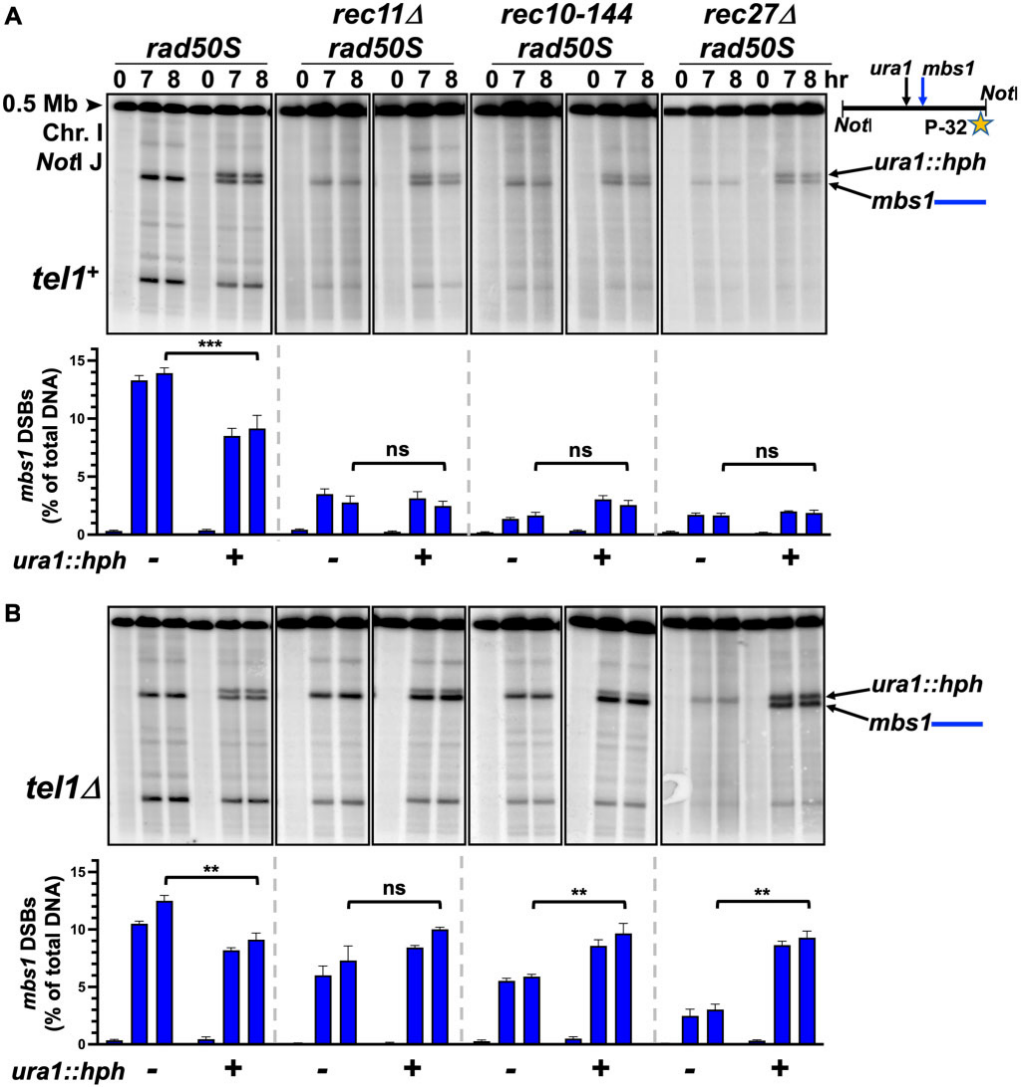
Meiotic DSB competition is impaired in cohesin and linear element mutants. A strong DSB hotspot (*ura1::hph*) was introduced 15 kb from the strong wild-type hotspot *mbs1* on Chromosome I to analyze the effect of cohesin and LinE mutations on DSB competition. Meiotic DNA was analyzed, by Southern blot hybridization, from *rec11*Δ, *rec10-144*, and *rec27*Δ strains, with *mbs1* only (left three lanes for each strain) or *mbs1* with *ura1::hph* (right three lanes), either with (**A**) or without (**B**) Tel1 present. The frequency of DSBs at *mbs1* is shown in the graphs (as % of total DNA). For each strain, the mean and SEM (or range) are shown from 2 to 7 experiments. The [^32^P]-labelled DNA probe was located on the right end of the 0.5 Mb NotI J fragment, as indicated by the star in scheme on the right. Two-tailed *P*-values calculated by unpaired *t*-test were ns (not significant, >.05), *(<.05), **(<.005), and ***(<.0005).

**Figure 3.**
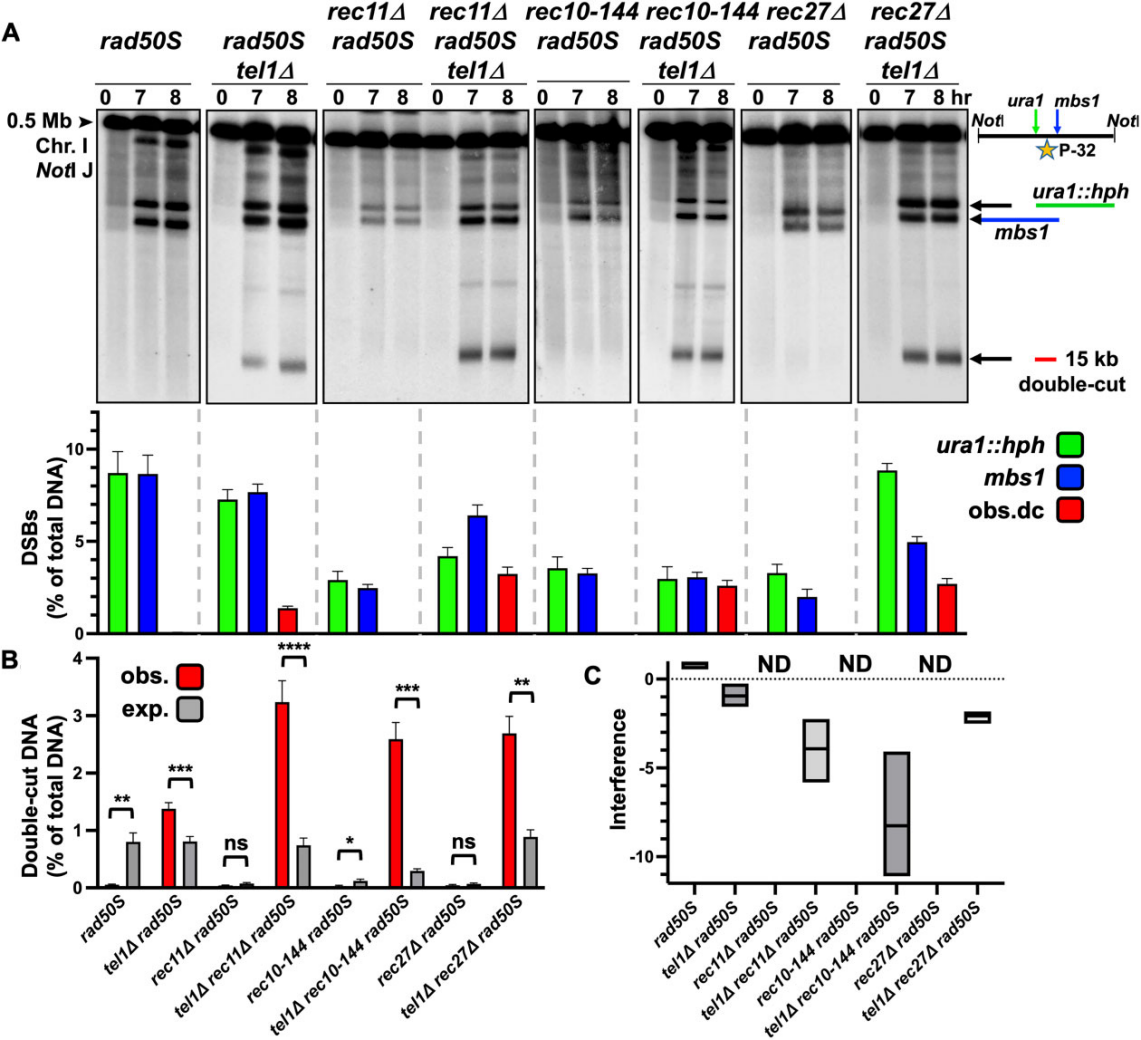
Frequent double-cut DNA and strong negative DSB interference are observed in cohesin and LinE *tel1Δ* double mutants. (**A**) The frequency of double-cut DNA was determined in the cohesin mutant *rec11*Δ and LinE mutants *rec10-144* and *rec27*Δ, in both *tel1^+^* and *tel1*Δ strains. DNA digested with NotI was separated by PFGE, blotted, and hybridized with a [^32^P]-labelled DNA probe (indicated by star on the right) located in the middle of the 0.5 Mb NotI J fragment, between the inserted DSB hotspot *ura1::hph* and *mbs1*, to detect DNA chromatids that were cut at both DSB hotspots. The mean DSB frequencies from the 7- and 8-h timepoints of *ura1::hph* DSBs (green), *mbs1* DSBs (blue), and observed DNA double-cuts at both *ura1::hph* and *mbs1* (red) are shown on the graph (as % of total DNA). (**B**) The double-cut frequencies are very low, and the observed (red) and expected (% *ura1::hph* x *% mbs1*; gray) double-cut frequencies (as % of total DNA) are replotted on a separate graph for clarity. Observed and expected values are the mean of the 7- and 8-h timepoints. For each strain, the mean and SEM are shown from three to six experiments. (**C**) DSB interference, calculated as 1 – (observed double-cut DNA/expected double-cut DNA), for each strain is plotted on the graph; the line in each box indicates the median value; the minimum and maximum values measured are at the bottom and top of each box. Interference was not determined (ND) for strains in which the expected double-cut DNA was <0.1%.

**Figure 4.**
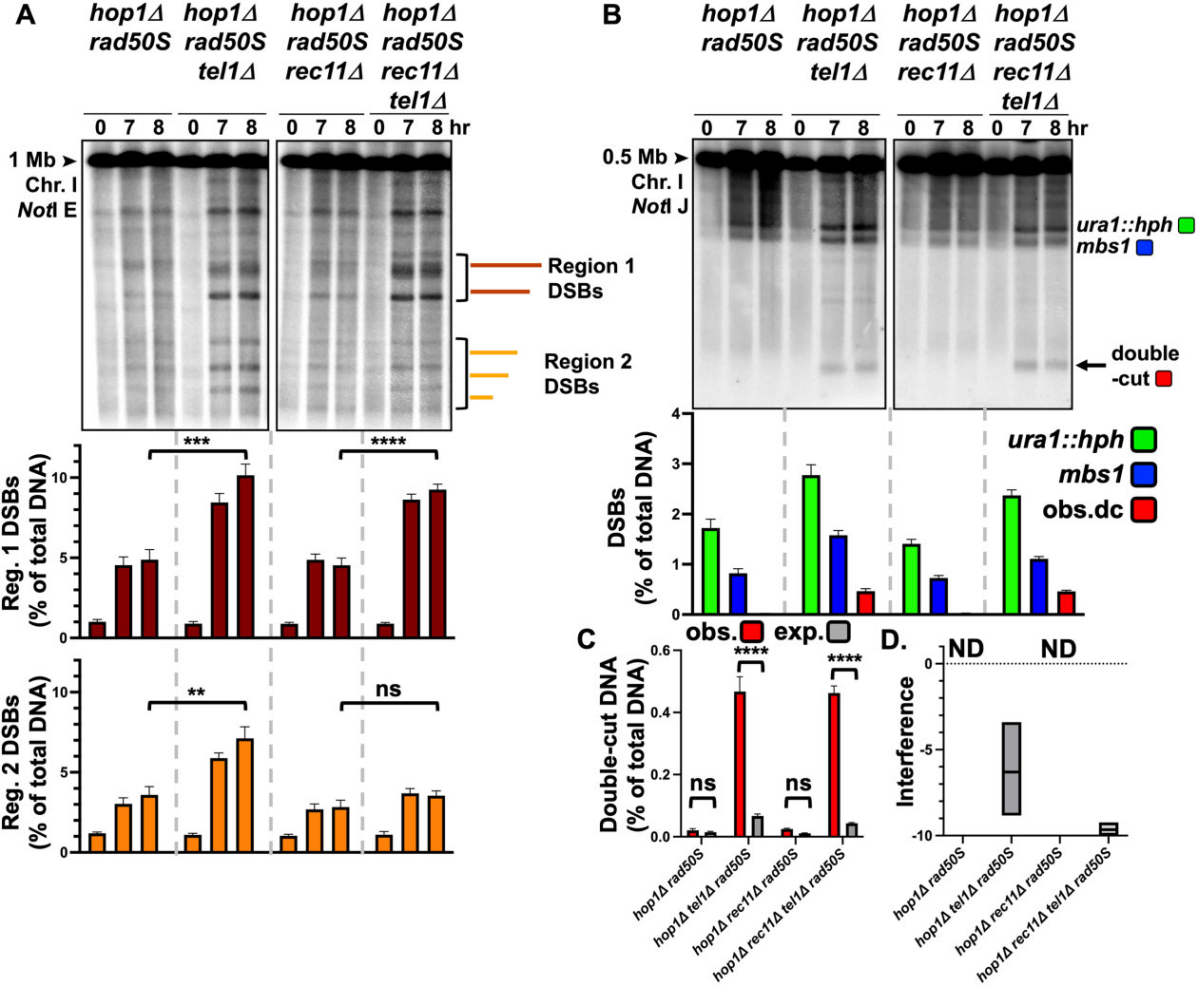
Loss of Hop1 leads to DSB repression by Tel1 and highly negative DSB interference without Tel1. (**A**) DSBs were measured in the HORMAD mutant *hop1*Δ *rad50S*, with and without Rec11 and Tel1. NotI-digested DNA was Southern blot-hybridized with a [^32^P]-labelled DNA probe on the left end of the 1 Mb NotI E fragment on Chromosome I (see Fig. 1), and DSBs were measured across an ~0.5 Mb (designated Regions 1 and 2). DSB frequencies (as % of total DNA) are represented in the graphs beneath the Southern blot images: upper graph, Region 1; lower graph, Region 2 (see Fig. 1 to compare with *hop1^+^*). For each strain, the mean and SEM are shown from 5–6 experiments. (**B**) The frequency of double-cut DNA was determined in *hop1*Δ *rad50S* and *hop1*Δ *rad50S rec11*Δ in both *tel1^+^* and *tel1*Δ strains. DNA digested with NotI was separated by PFGE, blotted, and hybridized with a [^32^P]-labelled DNA probe located in the middle of the 0.5 Mb NotI J fragment, between the inserted DSB hotspot *ura1::hph* and *mbs1*, to detect DNA chromatids that were cut at both DSB hotspots. The mean DSB frequencies from the 7- and 8-h timepoints of *ura1::hph* DSBs (green), *mbs1* DSBs (blue), and observed DNA double-cuts at both *ura1::hph* and *mbs1* (red) are shown on the graph (as % of total DNA). (**C**) The double-cut frequencies are very low, and the observed (red) and expected (% *ura1*::hph x *% mbs1*; gray) double-cut frequencies (as % of total DNA) are replotted on a separate graph for clarity. Observed and expected values are the mean of the 7- and 8-h timepoints. For each strain, the mean and SEM are shown from three to six experiments. (**D**) DSB interference, calculated as 1 – (observed double-cut DNA/expected double-cut DNA), for each strain is plotted on the graph; the line in each box indicates the median value; the minimum and maximum values measured are at the bottom and top of each box. Interference was not determined (ND) for strains in which the expected double-cut DNA was <0.1%.

**Figure 5.**
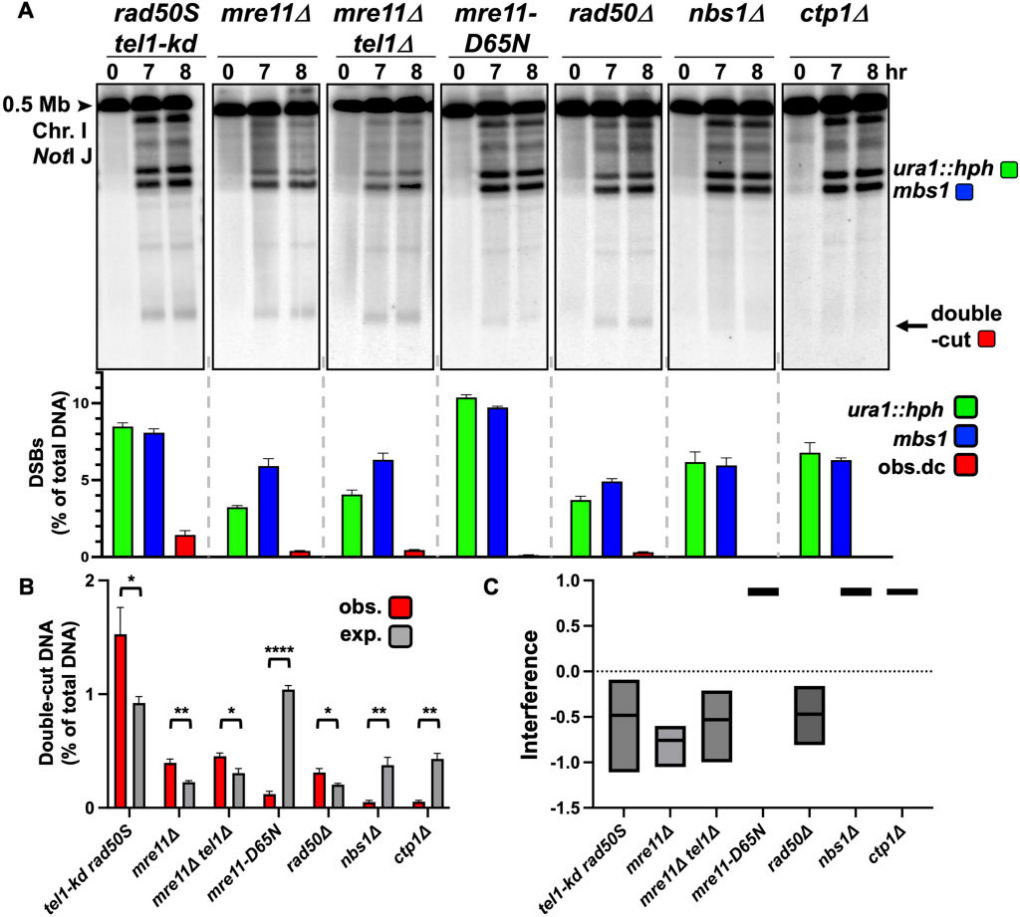
DSB interference is dependent on Mre11 and Rad50, but not Nbs1. (**A**) The frequency of double-cut DNA was detected in various mutants of the MRN DNA repair complex. NotI-digested DNA was Southern blot hybridized with a [^32^P]-labelled DNA probe located in the middle of the 0.5 Mb NotI J fragment (see Fig. 3), between the inserted DSB hotspot *ura1::hph* and *mbs1*, to detect DNA chromatids that were cut at both DSBs. The mean DSB frequencies from the 7- and 8-h timepoints of *ura1::hph* DSBs (green), *mbs1* DSBs (blue), and observed DNA double-cuts at both *ura1::hph* and *mbs1* (red) are shown on the graph (as % of total DNA). (**B**) The double-cut frequencies are very low, and the observed (red) and expected (% *ura1*::hph x *% mbs1*; gray) double-cut frequencies (as % of total DNA) are plotted on a separate graph for visibility. Observed and expected values are the mean of the 7- and 8-h timepoints. For each strain, the mean and SEM are shown from three or four experiments. (**C**) DSB interference, calculated as 1 – (observed double-cut DNA/expected double-cut DNA), for each strain is plotted on the graph; the line in each box indicates the median; the minimum and maximum values measured are at the bottom and top of each box.

**Figure 6.**
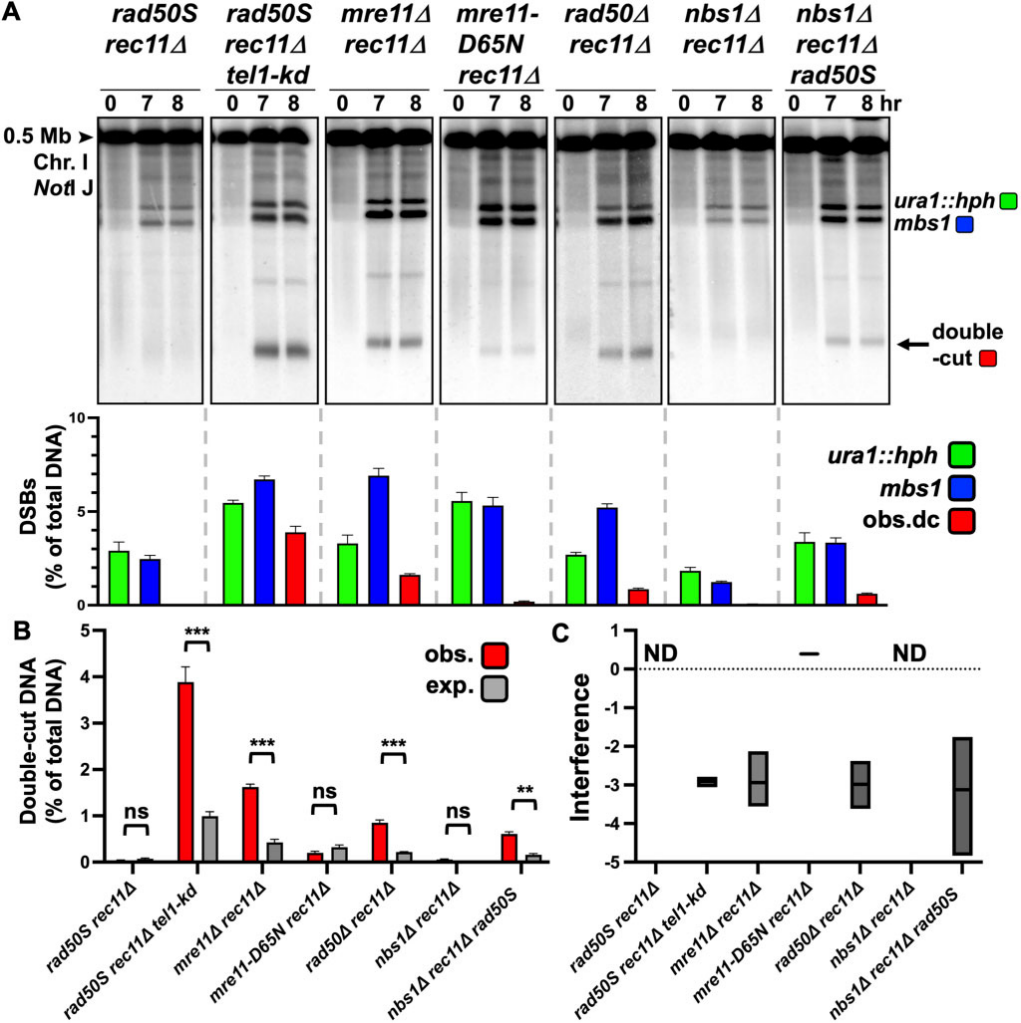
*mre11*Δ and *rad50*Δ, like *tel1*Δ, have strong negative DSB interference in double mutants with *rec11*Δ. (**A**) The frequency of double-cut DNA was detected in various mutants of the MRN DNA repair complex in a cohesin-deficient *rec11*Δ strain and a *rec11*Δ *tel1-kd* strain for comparison. NotI-digested DNA was Southern blot hybridized with a [^32^P]-labelled DNA probe located in the middle of the 0.5 Mb NotI J fragment (see Fig. 3), between the inserted DSB hotspot *ura1::hph* and *mbs1*, to detect DNA chromatids that were cut at both DSBs. The mean DSB frequencies from the 7- and 8-h timepoints of *ura1::hph* DSBs (green), *mbs1* DSBs (blue), and observed DNA double-cuts at both *ura1::hph* and *mbs1* (red). (**B**) The double-cut frequencies are very low, and the observed (red) and expected (% *ura1*::hph x *% mbs1*; gray) double-cut frequencies (as % of total DNA) are plotted on a separate graph for visibility. Observed and expected values are the mean of the 7-and 8-h timepoints. For each strain, the mean and SEM are shown from three or four experiments. (C) DSB interference, calculated as 1 – (observed double-cut DNA/expected double-cut DNA), for each strain is plotted on the graph. The line in each box indicates the median; the minimum and maximum values measured are at the bottom and top of each box. Interference was not determined (ND) for strains in which the expected double-cut DNA was <0.1%.

*Schizosaccharomyces pombe* strains were cultured and induced for meiosis as previously described [86]. Briefly, haploid *pat1-as* (L95G)::*kanMX6* strains were grown to saturation in 5 ml YEL at 30°C, then inoculated 1:100 into EMM2* and grown 2–3 days at 30°C. Cultures were diluted to OD_600_ of 0.1 in 125 ml EMM2* and grown at 25°C, with shaking, until OD_600_ reached 0.35–0.4 (~16–20 h, depending on the strain genotype). Cells were washed once and resuspended in 125 ml EMM2*–NH_4_Cl and incubated at 25°C, with shaking, for 18 h. The ATP analog inhibitor 3-MB-PP1 (Toronto Research Chemicals) was added to 25 µM to start synchronous meiosis. Thirty milliliters of cells were harvested at 0, 7, and 8 h and washed once in cold 50 mM EDTA at 4°C. The supernatant was removed, and the cell pellets were frozen in liquid nitrogen and stored at −80°C until DNA extraction. Cells were embedded in agarose plugs, and DNA was extracted in the plugs to prevent DNA breakage [86]. The agarose-embedded DNA was digested with NotI restriction enzyme, and the DNA separated by pulsed-gel electrophoresis in 0.8% Certified Megabase Agarose (Bio-Rad) in 0.5× TBE at 14°C. The electrophoresis conditions were 26 h at 6 V/cm with either a 7.9-s initial switch time to 54.2-s final switch time (linear ramp) for Fig. 2, or 2.2-s initial switch to 93.7-s final switch (linear ramp) for all other figures. Southern blot hybridization was as described [86]; oligos used for [^32^P]-labeled DNA probes are listed in Supplementary Table S2. The blots were scanned on a Typhoon Odyssey (GE Healthcare) and analyzed with ImageQuant XL (GE Healthcare) software. Quantifications of DSBs in Figs. 1, 2, and 4A and Supplementary Figs. S1, S3, S5, S6A, and S7 are the means of data at 0, 7, or 8 h. Quantifications of DSBs in Figs. 3, 4B, 5, and 6 and Supplementary Figs. S2, S4, S6B, and S8 are the means of data at 7 and 8 h combined.

### DSB interference calculation

DSB interference (I) was calculated in the same manner as genetic interference, using the equation I = 1 – (observed double-cut frequency/expected double-cut frequency), where expected double-cut frequency is (frequency of *ura1::hph* DSBs) × (frequency of *mbs1* DSBs). When double-cut DSBs (dc) were very frequent, the frequency of dc was added to both hotspots: I = 1- [obs. dc/(*ura1::hph* DSB + dc) x (*mbs1* DSB + dc)].

### Intragenic gene conversion assay

*Schizosaccharomyces pombe* strains with either *ura1::hph* or *ura1-171* alleles were mated, and the spores analyzed as described [85]. Briefly, 100 μl of each saturated YEL culture was harvested, mixed, washed three times in dH_2_O, and spotted on SPA with adenine and uracil (100 µg/ml), and incubated at 25°C for 3–4 days. Spores were harvested into 0.5 ml dH_2_O, glusulase (β-glucuronidase/arylsulfatase; Millipore Sigma**)** was added 1:100, and the suspension incubated at 30°C for 6 h. 0.5 ml of 60% EtOH was added, and the mixture incubated at room temperature for 15 min. Spores were washed three times in dH_2_O and resuspended in 1 ml of dH_2_O and stored at 4°C.

### *tel1-kd* strain construction

The 1.8-kb HindIII fragment containing the *ura4^+^* gene was amplified by polymerase chain reaction (PCR) from pYF20 with oligos OL5254 and OL5255 containing 80 nt DNA sequence from the Tel1 ORF (Supplementary Table S2), from nucleotides 7787–7866 and 7969–8048. Transformation of strain GP7080 with the PCR fragment generated a *tel1::ura4^+^* substitution at nucleotides 7867 to 7968 of the Tel1 ORF, verified by PCR, by selecting for Ura^+^ colonies, creating strain GP9400. Plasmid pFA6-*tel1-kd-natMX6* (created and sent to us by Dr. Israel Salguero in the lab of Dr. Stephen Jackson) was used as the template with oligos OL5256 and OL5257 to generate a PCR fragment with mutations G2629D, D2630A, N2635K, and D2649E at the active site kinase residues of Tel1, but without the linked *natMX6* cassette. This PCR product (see Supplementary Table S2) was used to transform strain GP9400 (*tel1::ura4^+^*) to *tel1-kd* by selecting for FOA-resistant colonies. The *tel1-kd* integration was verified by sequencing, creating strain GP9402.

### *hop1-5A* construction

We cloned the 1851 bp ORF of *hop1*, plus 433 bp upstream and 476 bp downstream, into pBR322, and, using site-directed mutagenesis with Q5 (New England Biolabs), we made the following nucleotide changes, with corresponding amino acid changes: T399G (S103A), T909G (S247A), T927G (S253A), A978G (T270A), and A1017G G1018C (S283A). The sequence was verified, and plasmid DNA was digested with Bpu10I and HaeII and used to transform GP565 *hop1::ura4^+^* to *hop1-5A* and FOA-resistance. The *hop1-5A* integration was verified by sequencing, creating strain GR570.

## Results

### Tel1 partially represses DSB formation in LinE and cohesin mutants; DSB competition depends on LinEs and cohesin

We analyzed meiosis in strains with the *pat1-as* (L95G) mutation, which allowed nitrogen-starved cells to be synchronously induced into meiosis at 25°C with the addition of an ATP-analog inhibitor (3MB-PP1) [87]. We could then measure the maximal DSB frequencies in strains with the MRN mutation *rad50S*, in which DSBs are not resected and thus accumulate [48, 88]. In the cohesin subunit mutant *rec11Δ* and the LinE mutants *rec10-144* (G727E) and *rec27Δ*, a three- to four-fold reduction of DSBs was seen at multiple hotspots (Fig. 1, designated Regions 1 and 2) across 0.5 Mb on Chromosome I. In double mutant strains with *tel1Δ*, Region 1 DSBs (upper graph) were significantly increased ~2-fold compared to the *tel1*^+^ single mutants but were still only 50% of wild-type. Region 2 DSBs (lower graph), however, were not significantly increased in the *tel1Δ* background, indicating this repression of DSBs by Tel1 is region (or site) specific. Similar results (Supplementary Fig. S1) were seen with another cohesin subunit mutant (*rec8Δ*) and LinE mutants (*rec25Δ* and *mug20Δ*). No DSBs were seen in a *rec12-164* (Y98F) *tel1Δ* mutant, as expected because Rec12 activity is essential for meiotic DSB formation [6, 37]. These results show that Tel1 partially prevents DSB formation in strains lacking intact cohesin and LinEs.

To determine whether these effects on DSB formation extend to recombination, we assayed intragenic gene conversion in the *ura1* gene. Note that *S. pombe* intragenic recombination occurs nearly exclusively by gene conversion [89, 90]. Crossing *ura1-61* and *ura1-171* generated 210 Ura^+^ spores/10^6^ total spores, in agreement with previous results [91]. Substitution of the first 1.8 kb of the *ura1* ORF with a hygromycin-resistance cassette [92], which removes the position of the *ura1-61* mutation, created a DSB and recombination hotspot (*ura1::hph*, see Fig. 2), as *ura1::hph* x *ura1-171* crosses generated 2400 Ura^+^ spores/10^6^ total spores (Table 1). *rec11Δ* and *rec10-144* mutations decreased *ura1* gene conversion (~2-fold versus *wt*), while double mutations with *tel1Δ* (and the triple mutant *rec11Δ rec10-144 tel1Δ*) increased Ura*^+^* frequency by 5-fold (~2.5-fold versus *wt*), reflecting the increased DSBs in these mutants (Fig. 1).

**Table 1.** *tel1*Δ increases meiotic recombination in cohesin and linear element mutants

Parental strains (*ura1::hph x ura1-171*)	Ura^+^/10^6^ viable spores	
	*tel1^+^*	*tel1Δ*
*rec^+^*	2400 ± 210 (7)	1700 ± 70 (5)
*rec11Δ*	960 ± 40 (6)	5000 ± 310 (6)
*rec10-144*	1200 ± 50 (4)	5000 ± 150 (4)
*rec10-144 rec11Δ*	1200 ± 290 (3)	4800 ± 830 (4)
*mbs1Δ*	3100 ± 150 (3)	4100 ± 550 (3)
*rec10-144 mbs1Δ*	1200 ± 110 (3)	2700 ± 300 (3)

Crosses were homozygous for the indicated mutations in the left column and for *tel1^+^* or *tel1Δ* as indicated in the middle and right columns; they were heterozygous for the indicated *ura1* mutations. Intragenic recombination without the DSB hotspot *ura1::hph* was measured by crossing *ura1-61* and *ura1-171*, which produced 210 ± 11 Ura^+^/10^6^ viable spores (*n *= 2), similar to results previously reported [91]. Data are the mean ± SEM from (*n*) crosses.

We next examined the phenomenon of DSB competition, in which addition of a strong DSB hotspot decreases the frequency of DSBs at a nearby hotspot (<200 kb away), as measured in a meiotic cell population [73, 80–84]. A LinE protein artificially tethered to a site creates a new DSB hotspot, but this hotspot did not impart competition on the surrounding DSBs [92]. This suggests that normal LinE loading, for example by cohesin, is important in establishing DSB competition. The inserted hotspot *ura1::hph* competes with the *mbs1* hotspot 15 kb away in wt (Fig. 2A; [92]), but the two hotspots showed no DSB competition in *rec11Δ, rec10-144*, or *rec27Δ* mutants (Fig. 2A); the *mbs1* DSB frequency was unchanged or slightly higher than without the inserted hotspot. The weak DSBs seen in these mutants might not be strong enough to establish competition, so we looked at DSBs in double mutants with *tel1Δ*; in previous assays, Tel1 did not affect DSB competition [72, 92], and our results here agree (Fig. 2B). However, none of the three *rec* mutants showed DSB competition, as the frequency of DSBs at *mbs1* was not reduced but rather was slightly increased in the presence of *ura1::hph* (Fig. 2B). These results show that DSB competition is dependent on cohesin and LinEs, and both must be in place for proper DSB regulation.

We deleted *mbs1* to determine if a competitive effect could be measured by the *ura1* genetic assay. When *mbs1* was deleted, the frequency of Ura^+^ recombinants in a *ura1-171* x *ura1::hph* cross was increased modestly in wild-type (∼30%) and strongly in *tel1Δ* (2.5-fold, Table 1), demonstrating competition between *mbs1* and the *ura1::hph* hotspot. Interestingly, the Ura^+^ frequency was reduced in *rec10-144 tel1Δ mbs1Δ* compared to *rec10-144 tel1Δ mbs1*^+^ (Table 1), in agreement with the DSB analysis: the presence of *ura1::hph* stimulates DSBs at *mbs1*, and *mbs1* stimulates *ura1^+^* recombination when cohesin and/or LinEs are absent.

### Loss of either cohesin or LinEs in *tel1Δ* mutants results in strong negative DSB interference

Next, we examined DSB interference by assaying the frequency of DNA cut at both the *mbs1* and *ura1::hph* hotspots, 15 kb apart. Like the wild-type strains, the *rec11Δ, rec10-144*, and *rec27Δ* mutants showed little double-cut DNA (Fig. 3A and B); however, the low frequency of DSBs and the very low expected frequency of double-cuts (<0.1%; Fig. 3B) did not allow reliable determination of interference (marked ND, Fig. 3C). However, when analyzed in a *tel1Δ* background, the frequency of double-cut DNA was remarkably high, even higher than with *tel1Δ* alone (∼3% versus 1.5%; Fig. 3A and B). DSB interference was also much more negative (−4, −8, and −2 in the three *tel1* double mutants versus −1 in the *tel1* single mutant, Fig. 3C), indicating that a DSB at one hotspot strongly stimulates a DSB at the second hotspot in the absence of both cohesin or LinEs and Tel1. Similar results (Supplementary Fig. S2A, B, and C) were seen with mutants of another cohesin subunit (*rec8Δ*) or LinEs (*rec25Δ* and *mug20Δ*), and in double cohesin-LinE mutants (*rec11Δ rec27Δ* and *rec11Δ rec10-144*), indicating cohesin and LinEs act together to control close DSBs.

### Loss of Hop1 leads to DSB repression by Tel1 (but not region-specific) and highly negative DSB interference without Tel1

DSBs and meiotic recombination are significantly decreased by the deletion of meiotic HORMAD protein *hop1Δ* in *S. pombe* [42, 93], so we questioned whether this was also due to Tel1 repression. As in cohesin and LinE mutants (Fig. 1), the low DSBs in Region 1 in *hop1Δ* increased ~2-fold when *tel1* was also deleted (Fig. 4A). Interestingly, the Region 2 DSBs also increased ~2-fold (Fig. 4A), which was not observed in cohesin or LinE mutants lacking Tel1 (Fig. 1). We next assayed double-cut DNA between the *ura1::hph* and *mbs1* DSB hotspots. There was no observable double-cut DNA in *hop1Δ*, though again the very low expected frequency of double-cuts (<0.1%; Fig. 4C) did not allow reliable determination of interference; significant double-cuts were observed in *hop1Δ tel1Δ* (Fig. 4B and C), which resulted in highly negative DSB interference (Fig. 4D). Similar results were seen when the cohesin subunit Rec11 was deleted in *hop1Δ* and *hop1Δ tel1Δ* strains (Fig. 4B, C, and D), which suggests that cohesin and Hop1 both act to control close DSBs. Since Hop1 is a direct target of Tel1 [78], we made a phosphorylation-minus mutant of Hop1; five potential S/TQ sites [94], the consensus for Tel1 (ATM) phosphorylation sites [95], were changed from serine to alanine. These mutations had no observed effect on DSB formation (Supplementary Fig. S3), and DSB interference was still strongly positive (Supplementary Fig. S4C), suggesting that Hop1 is not a direct target, or not the only target, of Tel1 to restrict DSBs.

### DSB interference depends on Mre11 and Rad50, but not on MRN-dependent resection

Recent studies have demonstrated that the MRN complex limits DSBs in mice [56], so we examined additional MRN mutants in *S. pombe rad50^+^* strains. (For the previous DNA analyses, the *rad50S* mutation was used to accumulate DSBs and thus enable their maximal quantification.) As expected, all MRN mutants examined, including deletions, accumulated DSBs (Supplementary Fig. S5), though with modest reductions in DSB frequencies. *rad50S* mutants have strong positive DSB interference (Fig. 3) [73], as do *nbs1Δ, mre11-D65N* (nuclease-minus), and *ctp1Δ* mutants (Fig. 5A, B, and C). In contrast, both *mre11Δ* and *rad50Δ* had negative interference like *tel1Δ* (Fig. 5). Since clipping of the Rec12-oligo and resection are impaired in all these strains [41, 42], active resection and repair are not necessary for positive DSB interference. *nbs1Δ* retained positive interference, and we thus infer that Mre11 and Rad50 are still present at a DSB during meiosis and can recruit Tel1 in the absence of Nbs1 in *S. pombe*. Negative DSB interference was also observed in *tel1-kd* (Fig. 5A, B, and C), in which the kinase active site was inactivated, evidence that the kinase activity of Tel1 is necessary for DSB interference. The *mre11Δ tel1Δ* mutant was like either single mutant (Fig. 5A, B, and C), which suggests MRN and Tel1 act coordinately after a DSB is made.

### DSB frequencies are similar in multiple MRN and NHEJ mutants

The similarities in phenotype of *tel1Δ, mre11Δ*, and *rad50Δ* suggest that MRN and Tel1 act together in response to DSBs. However, the *mre11Δ* and *rad50Δ* strains consistently had marginally fewer DSBs than a *rad50S* strain, and *mre11Δ tel1Δ* had fewer than *rad50S tel1Δ* (Fig. 1 and Supplementary Fig. S5). To address the possibility that some repair, and thus loss of observed DSBs, occurred through NHEJ in the absence of MRN, we studied *pku70Δ* derivatives, which lack NHEJ [96, 97]. *pku70Δ* had no significant effect on DSB formation or DSB interference in *rad50S, rad50S tel1Δ, mre11Δ*, or *mre11Δ tel1Δ* strains (Fig. 1 and Supplementary Fig. S6A and D). Therefore, NHEJ appears to be very infrequent in the absence of MRN during meiosis in *S. pombe*.

In vegetative cells, Tel1 is hyperactive in *rad50S* strains of *S. cerevisiae* and mice [98, 99]. There was no significant difference between *rad50S* strains with or without Tel1 (Figs. 1, 2, and 3A), but DSBs in the cohesin or LinE mutant *rad50S* backgrounds might be repressed by hyperactive Tel1. Therefore, it was necessary to assay DSBs in a different repair-deficient mutant, *mre11-D65N* [41, 42, 100]. When comparing the *rad50S* versus *mre11-D65N* derivatives, DSBs in Region 1 were increased significantly by *mre11-D65N*, ~2-fold, in *rec27Δ* (4.8 versus 9.2, *P *= .006, Fig. 1 and Supplementary Fig. S3), but not significantly increased in *rec11Δ* (7.6 versus 8.7, *P *= .57; Fig. 1 and Supplementary Fig. S7) or *hop1Δ* (4.9 versus 5.6, *P *= .55; Fig. 4A and Supplementary Fig. S3). Additionally, *rad50S nbs1Δ rec11Δ* triple mutant showed increased Region 1 DSBs compared to *nbs1Δ rec11Δ* (8.9 versus 4.9, *P *= .1; Supplementary Fig. S7), a characteristic of less Tel1 function and not hyperactivity caused by *rad50S*.

### Loss of cohesin in *mre11Δ* and *rad50Δ* mutants results in strong negative DSB interference

We found *mre11Δ rec11Δ* and *rad50Δ rec11Δ* mutants had a three- to four-fold increase of DNA double-cut at both *mbs1* and *ura1::hph* (Fig. 6A and B) compared to *mre11Δ rec11^+^* and *rad50Δ rec11^+^* (Fig. 5A and B), similar to observations in *tel1-kd rad50S rec11Δ* (Fig. 6A and B) compared to *tel1-kd rad50S rec11^+^* (Fig. 5A and B), and earlier results with *tel1Δ* (Fig. 3A and B). All three double mutants had highly negative DSB interference (Fig. 6A, B, and C). Conversely, none of the *rad50S rec11Δ, nbs1Δ rec11Δ*, or *mre11-D65N rec11Δ* double mutants produced double-cut DNA at high frequency (Fig. 6A and B). The low frequencies of DSBs in *rad50S rec11Δ* and *nbs1Δ rec11Δ* mutants and the expected infrequent double-cut DNA (< 0.1%) do not allow DSB interference to be calculated (ND, Fig. 6C), but the *mre11-D65N rec11Δ* mutant had weaker positive DSB interference compared to *mre11-D65N rec11^+^* (0.4 versus 0.9, Figs. 4C and 5C); the observed double-cut DNA was not significantly different than that expected in *mre11-D65N rec11Δ*, so it is unclear if there is weak positive or no interference. The *mre11-D65N rec27Δ* mutant did not show an interference decrease compared to *mre11-D65N rec27^+^* (0.9 versus 0.9; Fig. 4C and Supplementary Fig. S4C). The *rad50S nbs1Δ rec11Δ* triple mutant also had increased double-cut DNA compared to *rad50S rec11Δ* or *nbs1Δ rec11Δ * mutants and had highly negative DSB interference (Fig. 6A, B, and C). The *rad50S* and *nbs1Δ* mutations combined may create a less active and/or less stable MRN complex, a phenotype that is exacerbated by the removal of intact cohesin. If the *rad50S* mutation hyperactivated Tel1, there would be stronger interference and *less* double-cut DNA, not the observed increase, when *rad50S* is added to *nbs1Δ rec11Δ*.

## Discussion

Identifying the factors that influence meiotic DSB formation is essential for determining the mechanism of DSB control. The proper timing and placement of meiotic DSBs and the resulting recombination are highly dependent on protein complexes that organize and align chromosomes [25]. In all species studied, the chromosome axis, upon which the synaptonemal complex can be built, is essential for proper meiotic recombination, and meiotic cohesins are necessary for the establishment of the axis [10, 11, 23, 33–36]. Loss of chromosome axis proteins results in less frequent DSBs and recombination [17–20]. The fission yeast *S. pombe* linear elements with Hop1 form chromosome axis structures that are dependent on meiotic cohesin [10, 23, 35, 37]. Our results show that cells lacking cohesin, LinEs, or Hop1 have reduced DSBs by the action of Tel1 kinase (Figs. 1 and 4A), and in Tel1’s absence there is an increase in chromatids that are cut twice at nearby hotspots 15 kb apart. This suggests that proper loading and interaction of hotspot-bound LinEs and Hop1 are necessary for the formation of DSBs sensitive to competition and interference; in their absence, Tel1 is activated to repress some DSBs, particularly at nearby hotspots. Some DSB hotspots are completely dependent on cohesin and LinEs, but other DSBs are not, which indicates that some additional, unidentified factors are involved.

Previous studies demonstrated that crossovers are reduced in *S. pombe* cohesin, LinE, and Hop1 mutants, but not uniformly, as some genetic intervals have stronger reductions than others [17, 18, 28, 93, 94]. DSBs were not repressed by Tel1 at all hotspots, as some DSBs did not increase in frequency in cohesin or LinE *tel1Δ* double mutants; however, this was different in *hop1Δ*, as both Region 1 and Region 2 DSBs were increased by *tel1Δ*. The causes of the regional variability and potential differences among DSB hotspots are intriguing and remain to be determined. The previous conclusion, based on observations with the *pat1-114* Ts mutant at 34°C, that LinEs are required for DSBs and are determinants of hotspot position is not strictly correct—DSBs in some regions can be formed at 25°C, although at reduced levels in the absence of LinEs, and Tel1 prevents their formation at high levels (Fig. 1). This agrees with microscopy of GFP-tagged LinE proteins that show aberrant morphology at high temperature compared to 25°C [101]. Of note, MRN mutants *mre11Δ* and *rad50Δ* also show stronger reductions of DSBs at 34°C [53] than at 25°C (Supplementary Fig. S5). Previous experiments showed that wt meiosis did not differ in many aspects of DSB formation, repair, and spore viability at 25°C and 34°C [41, 53, 102, 103, 104], but the mutants used here are more sensitive to temperature changes.

We pursued other factors that act with Tel1 to limit DSBs. An obvious candidate was the MRN complex. During mitotic DNA damage, MRN recruits and activates Tel1, which in turn phosphorylates itself and MRN [62, 105]. This interaction creates a feedback loop that enables the signal cascade initiated by Tel1 phosphorylation of multiple proteins to delay the cell cycle until DNA damage is repaired. Because of the interdependent nature of MRN and Tel1, use of MRN mutants to accumulate DSBs is potentially problematic. Tel1 is hyperactive in mice and *S. cerevisiae rad50S* strains [98, 99], which could result in the low DSBs seen in *rad50S* cohesin and LinE mutants (Fig. 1 and Supplementary Fig. S1). *mre11-D65N rec11Δ* and *mre11-D65N hop1Δ* mutants did show small increases in DSBs compared to *rad50S rec11Δ* and *rad50S hop1Δ* that were statistically insignificant; however, there was a significant increase in DSBs in *rec27Δ mre11-D65N* compared to *rec27Δ rad50S. rad50S tel1Δ* did not significantly increase DSB frequencies in Region 1 or 2 (Fig. 1), or at *mbs1* or *ura1::hph* (Fig. 3A) compared to *rad50S tel1*^+^. Thus, while there is some evidence of Tel1 hyperactivation in *rad50S*, it is relatively minor, and *rad50S* and *mre11-D65N* both have equally strong positive DSB interference (Figs. 3C and 5C), suggesting similar Tel1 activity in response to meiotic DSBs. It is possible that other functions of Tel1 are affected differentially depending on the MRN mutant.

Recent studies of meiosis in budding yeast and mice have demonstrated Tel1 and MRN’s role in limiting multiple proximal DSBs (<1 kb apart) on the same chromatid, which can have deleterious effects [56, 67, 74–76]. Our results in *S. pombe* show that *mre11Δ* and *rad50Δ* result in phenotypes very similar to those of *tel1Δ*: wild-type positive DSB interference turns negative (Fig. 5), and DSBs are increased in cohesin mutant strains that lack MR (Mre11 or Rad50) (Fig. 6). However, these phenotypes are not displayed in MRN point mutants *rad50S* and *mre11-D65N* (nuclease-negative), *ctp1Δ*, or *nbs1Δ*,in which resection and clipping are impaired [106]. This suggests that MRN is first recruited to a DSB end and then recruits and activates Tel1 to stop further DBSs on the same DNA chromatid, independent of DNA resection; Tel1 is also recruited to mitotic DNA damage by nuclease-deficient MRN [107].

We were surprised that the *nbs1Δ* mutant still had positive DSB interference, as Nbs1 interacts directly with Tel1 [108], and mutants of Xrs2, the Nbs1 homolog, in budding yeast are defective in short-range (<1 kb) DSB interference [77]. Our results are consistent with studies in *S. pombe* that show MR alone is enough to recruit Tel1 to DSBs, as H2A phosphorylation by Tel1 was observed in *nbs1Δ*, but not in *mre11Δ*, mutants during mitotic DNA damage when Tel1 was overexpressed, and Mre11 is recruited to DSBs in the absence of Nbs1 [109]. Furthermore, Rec12-oligo clipping is partially active, and spore viability is higher, especially at 25°C, in *nbs1Δ* compared to *mre11Δ* or *rad50Δ* [41]. The *rad50S nbs1Δ rec11Δ* mutant had a significant increase in double-cut DNA that is indicative of no Tel1 action, but the *nbs1Δ rec11Δ* and *rad50S rec11Δ* mutants did not (Fig. 6A, B, and C). These results suggest an Mre11–Rad50 interaction with cohesin (or LinEs) and that this interaction is affected by the combination of *nbs1Δ * and *rad50S* mutations. LinE formation is dependent on the presence of MRN (but not resection), as nuclear structures seen by electron microscopy form normally in a *rad50S* mutant but not in a *rad50Δ* mutant [106], suggesting an MRN–LinE interaction. In cohesin and LinE mutants, MRN is likely present at the initial DSB site, and lack of a proper chromosome axis may trigger MRN to coordinate with Tel1 to repress DSB formation. How this interaction occurs is of interest but not yet known.

We have investigated double-cut DNA between DSB hotspots in *S. pombe* only at a relatively long distance (15–180 kb, Fig. 3 and Supplementary Fig. S8). It is not known if *S. pombe* shows the same frequent double-cutting within the same narrow DSB hotspot (35–200 bp) as seen in mice [56, 74]. The large amount of Spo11-oligos generated in an Mre11 conditional mutant in mice is not observed in any tested MRN mutant of *S. pombe* [41, 42, 106]. There is also a clear difference in the amount of excess DSBs prevented by Tel1—there is not a 10-fold increase in a *tel1Δ* mutant (Fig. 1) as seen in mice [65]. However, the loss of Mre11 in mice results in increased multiple cut chromatids, but loss of Mre11 nuclease activity does not [56]; this is similar to the phenotypes of the *S. pombe mre11* mutants analyzed here.

The robust increase of double-cut chromatids in strains lacking both the pre-DSB action of cohesin, LinEs, and Hop1 and the post-DSB action of MRN and Tel1 is very interesting, as the double-cuts are much more frequent than expected from random breakage (Figs. 3, 6 and Supplementary Fig. S2); there are multiple possibilities for the cause of this high negative DSB interference. Since DSBs are measured in a population, they could result from “hot cells,” a subset of cells that are more active and have excess DSB formation compared to the other cells. A hot cell is more likely to have multiple DSBs—a DSB at *mbs1* would frequently result in a second DSB at *ura1::hph* due to higher DSB activity in that “hot” cell. However, meiotic crossovers measured on two different *S. pombe* chromosomes occurred independently, providing evidence against hot cells in either *tel1^+^* or *tel1Δ* [73]. The negative DSB interference also decreases as the distance between hotspot pairs increases [73] (Supplementary Fig. S8), which is not predicted by hot cells. In budding yeast, it was proposed that some chromosomal domains have higher intrinsic DSB activity, which leads to DSB clusters, and observed negative interference that is eliminated when meiotic prophase is extended [110]. The study here is limited to only a few hotspot pairs (Supplementary Fig. S8), and this region may have higher DSB potential than other parts of the genome, and the large increases of double-cut DNA may not occur between all hotspots. We prefer the interpretation that cohesin, LinEs, and Hop1, in addition to being involved in the initiation of DSBs, have a role in inhibiting multiple DSBs, structurally or through additional factors, such as inhibiting the DSB-forming complex. MRN could also provide a surveillance function, not only for DSBs but also for chromosome axis components, to prevent additional DSBs on a disorganized DNA substrate.

**Figure 7.**
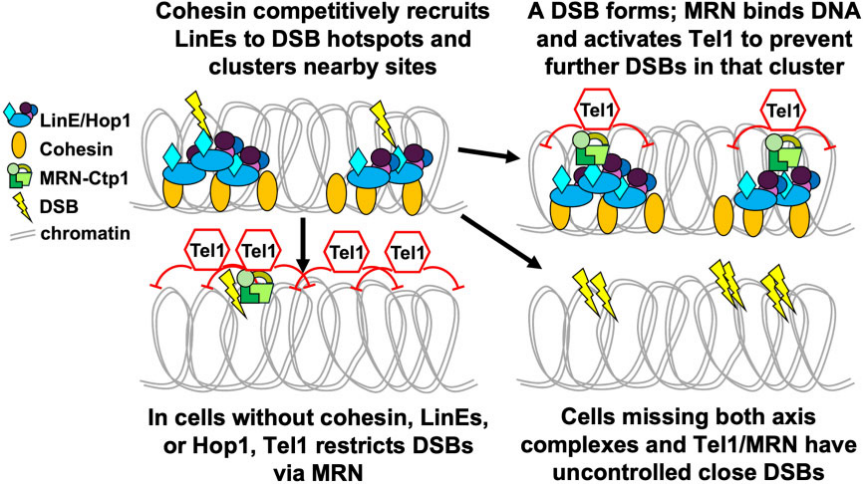
A model of DSB interference. Cohesin and the linear element complex cooperatively load onto meiotic DNA at sites of potential DSB hotspots. These protein complexes cluster together and bring the potential hotspots into physical proximity, allowing DSBs to be coordinately regulated over a limited distance. Cohesin, LinEs, and Hop1 recruit or activate the DSB-forming complex, and a DNA break is made (top left). In response to this break, the MRN-Ctp1 DNA repair complex is recruited to the DSB. We propose that MRN in turn recruits the Tel1 kinase, and Tel phosphorylates the DSB-forming complex to prevent further DSBs at other DSB hotspots in that cluster (top right). In the absence of MRN or Tel1, multiple DSBs are made preferentially in the same cluster, resulting in the observed negative DSB interference. In the absence of cohesin/LinEs, MRN and Tel are active and restrict some DSBs (bottom left). Without cohesin/LinEs/Hop1 (chromosome axis complexes) and MRN/Tel1, both the regulatory cluster and the inhibitory phosphorylation by Tel1 are absent, resulting in increased double-cut DNA and strong negative DSB interference (bottom right).

We propose that during *S. pombe* meiosis, the LinEs are loaded at a subset of potential DSB hotspots through Rec10’s interaction with phosphorylated cohesin subunit Rec11 ([21, 22] (Fig. 7). LinE-bound sites (DSB hotspots) are brought together through an undetermined mechanism, perhaps by the cohesin complex forming clusters at the same time as loading LinEs. Recent results *in vitro* with purified LinE complex also suggest that this could happen through phase-separated condensation of the LinE complex [111]. The *in vitro* purified LinE protein condensates form more readily when a LinE component (Rec27) can bind DNA. Self-assembly and condensation have been proposed and demonstrated for complexes of the SC and DSB complex in other species and proposed as a mechanism of DSB and crossover control [112, 113]. The proper interactions of the LinE proteins (and likely Hop1) with DNA, and subsequently with other close DNA-bound LinE complexes, are an important mechanism of DSB control in *S. pombe*. We do note, however, that because MRN is necessary for DSB DNA end tethering [114, 115] promoted by Tel1 [116], destabilized DNA ends in MRN- or Tel1-deficient strains conceivably could influence multiple DSBs on the same chromatids. When the cohesin or LinE complex is also missing and the chromosome axis structure is chaotic, perhaps the DSB is no longer recognized and multiple cuts are made.

The loss of DSB competition in cells that lack either cohesin or LinEs (Fig. 2) and the high frequency of double-cut chromatids (Fig. 3) support a shared function in regulating DSBs spatially. The proposed clustering of LinEs initially occurs over 100–200 kb (∼20–40 cM), corresponding to the extent of DSB competition and interference [73] (Supplementary Fig. S8). This may result from LinEs and Hop1 interacting over the limited extent of chromatin loops within the Rec8 cohesin-mediated axis (via Rec11) [117]. In this scenario, a DSB is made, causing localization of MRN and Tel1, which then prevents more DSBs in the clustered interval (Fig. 7). In strains lacking cohesins (or LinEs or Hop1) and Tel1, close DSB hotspots are unrestricted and cooperatively loaded with DSB complexes and then broken at nearly the same time. The targets of Tel1 after DSB formation in *S. pombe* remain to be determined. In a phospho-proteomics study of meiotic *S. cerevisiae* cells, 19% of the DSB-dependent phosphorylation sites detected had the Tel1 consensus motif [118], and evidence suggests that the Rec114–Mei4–Mer2 complex, related to *S. pombe* Rec15–Rec7–Rec24, is one potential target [68, 119]. The requirement for Tel1 to limit meiotic DSBs appears to be conserved. Perhaps additional features of this mechanism are conserved as well, and the results reported here may apply to other species.

## Supplementary Material

gkaf1405_Supplemental_File

## Data Availability

All data are contained within the manuscript and/or supplementary files.

## References

[1] Jones G, Kleckner N, Zickler D. Meiosis through three centuries. Chromosoma. 2024;133:93–115. 10.1007/s00412-024-00822-0. 38730132 PMC11180163

[2] Raghavan AR, Hochwagen A. Keeping it safe: control of meiotic chromosome breakage. Trends Genet. 2024;41:315–29. 39672680 10.1016/j.tig.2024.11.006 PMC11981862

[3] Arter M, Keeney S. Divergence and conservation of the meiotic recombination machinery. Nat Rev Genet. 2024;25:309–25. 10.1038/s41576-023-00669-8. 38036793

[4] Bergerat A, de Massy B, Gadelle D et al. An atypical topoisomerase II from archaea with implications for meiotic recombination. Nature. 1997;386:414–7. 10.1038/386414a0. 9121560

[5] Keeney S, Giroux CN, Kleckner N. Meiosis-specific DNA double-strand breaks are catalyzed by Spo11, a member of a widely conserved protein family. Cell. 1997;88:375–84. 10.1016/S0092-8674(00)81876-0. 9039264

[6] Cervantes MD, Farah JA, Smith GR. Meiotic DNA breaks associated with recombination in *S. pombe*. Mol Cell. 2000;5:883–8. 10.1016/S1097-2765(00)80328-7. 10882124

[7] Zheng Z, Zheng L, Arter M et al. Reconstitution of SPO11-dependent double-strand break formation. Nature. 2025;639:784–91. 10.1038/s41586-025-08601-2. 39972129 PMC11922745

[8] Oger C, Claeys Bouuaert C. SPO11 dimers are sufficient to catalyse DNA double-strand breaks *in vitro*. Nature. 2025;639:792–9. 10.1038/s41586-024-08574-8. 39972130 PMC11922746

[9] Tang X, Hu Z, Ding J et al. In vitro reconstitution of meiotic DNA double-strand-break formation. Nature. 2025;639:800–7. 10.1038/s41586-024-08551-1. 39972125 PMC11922769

[10] Molnar M, Bahler J, Sipiczki M et al. The *rec8* gene of *Schizosaccaromyces pombe* is involved in linear element formation, chromosome pairing and sister-chromatid cohesion during meiosis. Genetics. 1995;141:61–73. 10.1093/genetics/141.1.61. 8536990 PMC1206740

[11] Klein F, Mahr P, Galova M et al. A central role for cohesins in sister chromatid cohesion, formation of axial elements, and recombination during yeast meiosis. Cell. 1999;98:91–103. 10.1016/S0092-8674(00)80609-1. 10412984

[12] Pasierbek P, Jantsch M, Melcher M et al. A *Caenorhabditis elegans* cohesion protein with functions in meiotic chromosome pairing and disjunction. Genes Dev. 2001;15:1349–60. 10.1101/gad.192701. 11390355 PMC312707

[13] Parisi S, McKay MJ, Molnar M et al. Rec8p, a meiotic recombination and sister chromatid cohesion phosphoprotein of the Rad21p family conserved from fission yeast to humans. Mol Cell Biol. 1999;19:3515–28. 10.1128/MCB.19.5.3515. 10207075 PMC84144

[14] Michaelis C, Ciosk R, Nasmyth K. Cohesins: chromosomal proteins that prevent premature separation of sister chromatids. Cell. 1997;91:35–45. 10.1016/S0092-8674(01)80007-6. 9335333

[15] Watanabe Y, Nurse P. Cohesin Rec8 is required for reductional chromosome segregation at meiosis. Nature. 1999;400:461–4. 10.1038/22774. 10440376

[16] Prieto I, Suja JA, Pezzi N et al. Mammalian STAG3 is a cohesin specific to sister chromatid arms in meiosis I. Nat Cell Biol. 2001;3:761–6. 10.1038/35087082. 11483963

[17] DeVeaux LC, Smith GR. Region-specific activators of meiotic recombination in *Schizosaccharomyces pombe*. Genes Dev. 1994;8:203–10. 10.1101/gad.8.2.203. 8299939

[18] Ellermeier C, Smith GR. Cohesins are required for meiotic DNA breakage and recombination in *Schizosaccharomyces pombe*. Proc Natl Acad Sci USA. 2005;102:10952–7. 10.1073/pnas.0504805102. 16043696 PMC1182449

[19] Schwacha A, Kleckner N. Interhomolog bias during meiotic recombination: meiotic functions promote a highly differentiated interhomolog-only pathway. Cell. 1997;90:1123–35. 10.1016/S0092-8674(00)80378-5. 9323140

[20] Mao-Draayer Y, Galbraith AM, Pittman DL et al. Analysis of meiotic recombination pathways in the yeast *Saccharomyces cerevisiae*. Genetics. 1996;144:71–86. 10.1093/genetics/144.1.71. 8878674 PMC1207519

[21] Sakuno T, Watanabe Y. Phosphorylation of cohesin Rec11/SA3 by casein kinase 1 promotes homologous recombination by assembling the meiotic chromosome axis. Dev Cell. 2015;32:220–30. 10.1016/j.devcel.2014.11.033. 25579976

[22] Phadnis N, Cipak L, Polakova S et al. Casein kinase 1 and phosphorylation of cohesin subunit Rec11 (SA3) promote meiotic recombination through linear element formation. PLoS Genet. 2015;11:e1005225. 10.1371/journal.pgen.1005225. 25993311 PMC4439085

[23] Lorenz A, Wells JL, Pryce DW et al. *S. pombe* meiotic linear elements contain proteins related to synaptonemal complex components. J Cell Sci. 2004;117:3343–51. 10.1242/jcs.01203. 15226405

[24] Kariyazono R, Oda A, Yamada T et al. Conserved HORMA domain-containing protein Hop1 stabilizes interaction between proteins of meiotic DNA break hotspots and chromosome axis. Nucleic Acids Res. 2019;47:10166–80. 10.1093/nar/gkz754. 31665745 PMC6821256

[25] Ur SN, Corbett KD. Architecture and dynamics of meiotic chromosomes. Annu Rev Genet. 2021;55:497–526. 10.1146/annurev-genet-071719-020235. 34530636

[26] Shodhan A, Xaver M, Wheeler D et al. Turning coldspots into hotspots: targeted recruitment of axis protein Hop1 stimulates meiotic recombination in *Saccharomyces cerevisiae*. Genetics. 2022;222: iyac106. 10.1093/genetics/iyac106. 35876814 PMC9434160

[27] Bähler J, Wyler T, Loidl J et al. Unusual nuclear structures in meiotic prophase of fission yeast: a cytological analysis. J Cell Biol. 1993;121:241–56. 10.1083/jcb.121.2.241. 8468345 PMC2200093

[28] Davis L, Rozalén AE, Moreno S et al. Rec25 and Rec27, novel components of meiotic linear elements, link cohesin to DNA breakage and recombination in fission yeast. Curr Biol. 2008;18:849–54. 10.1016/j.cub.2008.05.025. 18514516 PMC3119532

[29] Estreicher A, Lorenz A, Loidl J. Mug20, a novel protein associated with linear elements in fission yeast meiosis. Curr Genet. 2012;58:119–27. 10.1007/s00294-012-0369-3. 22362333 PMC3310140

[30] Page SL, Hawley RS. The genetics and molecular biology of the synaptonemal complex. Annu Rev Cell Dev Biol. 2004;20:525–58. 10.1146/annurev.cellbio.19.111301.155141. 15473851

[31] Ding DQ, Matsuda A, Okamasa K et al. Linear elements are stable structures along the chromosome axis in fission yeast meiosis. Chromosoma. 2021;130:149–62. 10.1007/s00412-021-00757-w. 33825974 PMC8426239

[32] West AM, Rosenberg SC, Ur SN et al. A conserved filamentous assembly underlies the structure of the meiotic chromosome axis. eLife. 2019;8:e40372. 10.7554/eLife.40372. 30657449 PMC6349405

[33] Blat Y, Protacio RU, Hunter N et al. Physical and functional interactions among basic chromosome organizational features govern early steps of meiotic chiasma formation. Cell. 2002;111:791–802. 10.1016/S0092-8674(02)01167-4. 12526806

[34] Pelttari J, Hoja MR, Yuan L et al. A meiotic chromosomal core consisting of cohesin complex proteins recruits DNA recombination proteins and promotes synapsis in the absence of an axial element in mammalian meiotic cells. Mol Cell Biol. 2001;21:5667–77. 10.1128/MCB.21.16.5667-5677.2001. 11463847 PMC87287

[35] Molnar M, Doll E, Yamamoto A et al. Linear element formation and their role in meiotic sister chromatid cohesion and chromosome pairing. J Cell Sci. 2003;116:1719–31. 10.1242/jcs.00387. 12665553

[36] Eijpe M, Offenberg H, Jessberger R et al. Meiotic cohesin REC8 marks the axial elements of rat synaptonemal complexes before cohesins SMC1β and SMC3. J Cell Biol. 2003;160:657–70. 10.1083/jcb.200212080. 12615909 PMC2173354

[37] Fowler KR, Gutiérrez-Velasco S, Martín-Castellanos C et al. Protein determinants of meiotic DNA break hotspots. Mol Cell. 2013;49:983–96. 10.1016/j.molcel.2013.01.008. 23395004 PMC3595357

[38] Mimitou EP, Symington LS. Nucleases and helicases take center stage in homologous recombination. Trends Biochem Sci. 2009;34:264–72. 10.1016/j.tibs.2009.01.010. 19375328

[39] Cejka P, Symington LS. DNA end resection: mechanism and control. Annu Rev Genet. 2021;55:285–307. 10.1146/annurev-genet-071719-020312. 34813349

[40] Neale MJ, Pan J, Keeney S. Endonucleolytic processing of covalent protein-linked DNA double-strand breaks. Nature. 2005;436:1053–7. 10.1038/nature03872. 16107854 PMC1262668

[41] Milman N, Higuchi E, Smith GR. Meiotic DNA double-strand break repair requires two nucleases, MRN and Ctp1, to produce a single size class of Rec12 (Spo11)-oligonucleotide complexes. Mol Cell Biol. 2009;29:5998–6005. 10.1128/MCB.01127-09. 19752195 PMC2772569

[42] Rothenberg M, Kohli J, Ludin K. Ctp1 and the MRN-complex are required for endonucleolytic Rec12 removal with release of a single class of oligonucleotides in fission yeast. PLoS Genet. 2009;5:e1000722. 10.1371/journal.pgen.1000722. 19911044 PMC2768786

[43] Mimitou EP, Symington LS. Sae2, Exo1 and Sgs1 collaborate in DNA double-strand break processing. Nature. 2008;455:770–4. 10.1038/nature07312. 18806779 PMC3818707

[44] Nicolette ML, Lee K, Guo Z et al. Mre11-Rad50-Xrs2 and Sae2 promote 5′ strand resection of DNA double-strand breaks. Nat Struct Mol Biol. 2010;17:1478–85. 10.1038/nsmb.1957. 21102445 PMC3059534

[45] Zakharyevich K, Ma Y, Tang S et al. Temporally and biochemically distinct activities of Exo1 during meiosis: double-strand break resection and resolution of double Holliday junctions. Mol Cell. 2010;40:1001–15. 10.1016/j.molcel.2010.11.032. 21172664 PMC3061447

[46] Mimitou EP, Yamada S, Keeney S. A global view of meiotic double-strand break end resection. Science. 2017;355:40–5. 10.1126/science.aak9704. 28059759 PMC5234563

[47] Brown MS, Bishop DK. DNA strand exchange and RecA homologs in meiosis. Cold Spring Harb Perspect Biol. 2015;7:a016659. 10.1101/cshperspect.a016659. PMC4292170 25475089

[48] Alani E, Padmore R, Kleckner N. Analysis of wild-type and *rad50* mutants of yeast suggests an intimate relationship between meiotic chromosome synapsis and recombination. Cell. 1990;61:419–36. 10.1016/0092-8674(90)90524-I. 2185891

[49] Johzuka K, Ogawa H. Interaction of Mre11 and Rad50: two proteins required for DNA repair and meiosis-specific double-strand break formation in *Saccharomyces cerevisiae*. Genetics. 1995;139:1521–32. 10.1093/genetics/139.4.1521. 7789757 PMC1206481

[50] Shima H, Suzuki M, Shinohara M. Isolation and characterization of novel *xrs2* mutations in *Saccharomyces cerevisiae*. Genetics. 2005;170:71–85. 10.1534/genetics.104.037580. 15716496 PMC1449720

[51] Chin GM, Villeneuve AM. *C. elegans mre-11* is required for meiotic recombination and DNA repair but is dispensable for the meiotic G(2) DNA damage checkpoint. Genes Dev. 2001;15:522–34. 10.1101/gad.864101. 11238374 PMC312651

[52] Borde V, Lin W, Novikov E et al. Association of Mre11p with double-strand break sites during yeast meiosis. Mol Cell. 2004;13:389–401. 10.1016/S1097-2765(04)00034-6. 14967146

[53] Young JA, Hyppa RW, Smith GR. Conserved and nonconserved proteins for meiotic DNA breakage and repair in yeasts. Genetics. 2004;167:593–605. 10.1534/genetics.103.023762. 15238514 PMC1470912

[54] Puizina J, Siroky J, Mokros P et al. Mre11 deficiency in Arabidopsis is associated with chromosomal instability in somatic cells and Spo11-dependent genome fragmentation during meiosis. Plant Cell. 2004;16:1968–78. 10.1105/tpc.104.022749. 15258261 PMC519189

[55] Lukaszewicz A, Howard-Till RA, Novatchkova M et al. MRE11 and COM1/SAE2 are required for double-strand break repair and efficient chromosome pairing during meiosis of the protist Tetrahymena. Chromosoma. 2010;119:505–18. 10.1007/s00412-010-0274-9. 20422424

[56] Kim S, Yamada S, Li T et al. Mouse MRE11-RAD50-NBS1 is needed to start and extend meiotic DNA end resection. Nat Commun. 2025;16:3613. 10.1038/s41467-025-57928-x. 40240347 PMC12003770

[57] Acharya SN, Many AM, Schroeder AP et al. *Coprinus cinereus rad50* mutants reveal an essential structural role for Rad50 in axial element and synaptonemal complex formation, homolog pairing and meiotic recombination. Genetics. 2008;180:1889–907. 10.1534/genetics.108.092775. 18940790 PMC2600930

[58] Keeney S . Spo11 and the formation of DNA double-strand breaks in meiosis. In: Egel R, Lankenau D-H (eds.), Recombination and Meiosis: Crossing-Over and Disjunction. Berlin: Springer, 2007, 81–123. 10.1007/7050_2007_026 PMC3172816 21927624

[59] Symington LS, Gautier J. Double-strand break end resection and repair pathway choice. Annu Rev Genet. 2011;45:247–71. 10.1146/annurev-genet-110410-132435. 21910633

[60] Shibata A, Jeggo P, Lobrich M. The pendulum of the Ku-Ku clock. DNA Repair. 2018;71:164–71. 10.1016/j.dnarep.2018.08.020. 30177438

[61] Lascarez-Lagunas LI, Nadarajan S, Martinez-Garcia M et al. ATM/ATR kinases link the synaptonemal complex and DNA double-strand break repair pathway choice. Curr Biol. 2022;32:4719–26. 10.1016/j.cub.2022.08.081. 36137547 PMC9643613

[62] Paull TT . Mechanisms of ATM activation. Annu Rev Biochem. 2015;84:711–38. 10.1146/annurev-biochem-060614-034335. 25580527

[63] Cooper TJ, Wardell K, Garcia V et al. Homeostatic regulation of meiotic DSB formation by ATM/ATR. Exp Cell Res. 2014;329:124–31. 10.1016/j.yexcr.2014.07.016. 25116420

[64] Lukaszewicz A, Lange J, Keeney S et al. Control of meiotic double-strand-break formation by ATM: local and global views. Cell Cycle. 2018;17:1155–72. 10.1080/15384101.2018.1464847. 29963942 PMC6110601

[65] Lange J, Pan J, Cole F et al. ATM controls meiotic double-strand-break formation. Nature. 2011;479:237–40. 10.1038/nature10508. 22002603 PMC3213282

[66] Joshi N, Brown MS, Bishop DK et al. Gradual implementation of the meiotic recombination program via checkpoint pathways controlled by global DSB levels. Mol Cell. 2015;57:797–811. 10.1016/j.molcel.2014.12.027. 25661491 PMC4392720

[67] Garcia V, Gray S, Allison RM et al. Tel1(ATM)-mediated interference suppresses clustered meiotic double-strand-break formation. Nature. 2015;520:114–8. 10.1038/nature13993. 25539084 PMC7116500

[68] Carballo JA, Panizza S, Serrentino ME et al. Budding yeast ATM/ATR control meiotic double-strand break (DSB) levels by down-regulating Rec114, an essential component of the DSB-machinery. PLoS Genet. 2013;9:e1003545. 10.1371/journal.pgen.1003545. 23825959 PMC3694840

[69] Joyce EF, Pedersen M, Tiong S et al. Drosophila ATM and ATR have distinct activities in the regulation of meiotic DNA damage and repair. J Cell Biol. 2011;195:359–67. 10.1083/jcb.201104121. 22024169 PMC3206348

[70] Kurzbauer MT, Janisiw MP, Paulin LF et al. ATM controls meiotic DNA double-strand break formation and recombination and affects synaptonemal complex organization in plants. Plant Cell. 2021;33:1633–56. 10.1093/plcell/koab045. 33659989 PMC8254504

[71] Lange J, Yamada S, Tischfield SE et al. The landscape of mouse meiotic double-strand break formation, processing, and repair. Cell. 2016;167:695–708. 10.1016/j.cell.2016.09.035. 27745971 PMC5117687

[72] Mohibullah N, Keeney S. Numerical and spatial patterning of yeast meiotic DNA breaks by Tel1. Genome Res. 2017;27:278–88. 10.1101/gr.213587.116. 27923845 PMC5287233

[73] Fowler KR, Hyppa RW, Cromie GA et al. Physical basis for long-distance communication along meiotic chromosomes. Proc Natl Acad Sci USA. 2018;115:E9333–42. 10.1073/pnas.1801920115. 30217891 PMC6176642

[74] Lukaszewicz A, Lange J, Keeney S et al. *De novo* deletions and duplications at recombination hotspots in mouse germlines. Cell. 2021;184:5970–84. 10.1016/j.cell.2021.10.025. 34793701 PMC8616837

[75] Prieler S, Chen D, Huang L et al. Spo11 generates gaps through concerted cuts at sites of topological stress. Nature. 2021;594:577–82. 10.1038/s41586-021-03632-x. 34108684

[76] Johnson D, Crawford M, Cooper T et al. Concerted cutting by Spo11 illuminates meiotic DNA break mechanics. Nature. 2021;594:572–6. 10.1038/s41586-021-03389-3. 34108687 PMC7611867

[77] Dorme M, Aithal R, Cayrou C et al. Xrs2 C-terminus mediates Tel1-dependent meiotic double-strand break interference. PLoS Genet. 2025;21:e1011904. 10.1371/journal.pgen.1011904. 41248173 PMC12622785

[78] Carballo JA, Johnson AL, Sedgwick SG et al. Phosphorylation of the axial element protein Hop1 by Mec1/Tel1 ensures meiotic interhomolog recombination. Cell. 2008;132:758–70. 10.1016/j.cell.2008.01.035. 18329363

[79] Ho HC, Burgess SM. Pch2 acts through Xrs2 and Tel1/ATM to modulate interhomolog bias and checkpoint function during meiosis. PLoS Genet. 2011;7:e1002351. 10.1371/journal.pgen.1002351. 22072981 PMC3207854

[80] Wu TC, Lichten M. Factors that affect the location and frequency of meiosis-induced double-strand breaks in *Saccharomyces cerevisiae*. Genetics. 1995;140:55–66. 10.1093/genetics/140.1.55. 7635308 PMC1206571

[81] Xu L, Kleckner N. Sequence non-specific double-strand breaks and interhomolog interactions prior to double-strand break formation at a meiotic recombination hot spot in yeast. EMBO J. 1995;14:5115–28. 10.1002/j.1460-2075.1995.tb00194.x. 7588640 PMC394615

[82] Jessop L, Allers T, Lichten M. Infrequent co-conversion of markers flanking a meiotic recombination initiation site in *Saccharomyces cerevisiae*. Genetics. 2005;169:1353–67. 10.1534/genetics.104.036509. 15654098 PMC1449552

[83] Robine N, Uematsu N, Amiot F et al. Genome-wide redistribution of meiotic double-strand breaks in *Saccharomyces cerevisiae*. Mol Cell Biol. 2007;27:1868–80. 10.1128/MCB.02063-06. 17189430 PMC1820458

[84] Fukuda T, Kugou K, Sasanuma H et al. Targeted induction of meiotic double-strand breaks reveals chromosomal domain-dependent regulation of Spo11 and interactions among potential sites of meiotic recombination. Nucleic Acids Res. 2008;36:984–97. 10.1093/nar/gkm1082. 18096626 PMC2241902

[85] Smith GR . Genetic analysis of meiotic recombination in *Schizosaccharomyces pombe*. Methods Mol Biol. 2009;557:65–76. 19799177 10.1007/978-1-59745-527-5_6 PMC2758532

[86] Hyppa RW, Smith GR. Using *Schizosaccharomyces pombe* Meiosis To Analyze DNA Recombination Intermediates. Methods Mol Biol. 2009;557:235–52. 19799186 10.1007/978-1-59745-527-5_15 PMC2758538

[87] Guerra-Moreno A, Alves-Rodrigues I, Hidalgo E et al. Chemical genetic induction of meiosis in *Schizosaccharomyces pombe*. Cell Cycle. 2012;11:1621–5. 10.4161/cc.20051. 22456336

[88] Young JA, Schreckhise RW, Steiner WW et al. Meiotic recombination remote from prominent DNA break sites in *S. pombe*. Mol Cell. 2002;9:253–63. 10.1016/S1097-2765(02)00452-5. 11864600

[89] Gutz H . Site specific induction of gene conversion in *Schizosaccharomyces pombe*. Genetics. 1971;69:317–37. 10.1093/genetics/69.3.317. 17248549 PMC1212708

[90] Brown SD, Mpaulo SJ, Asogwa MN et al. DNA sequence differences are determinants of meiotic recombination outcome. Sci Rep. 2019;9:16446. 10.1038/s41598-019-52907-x. 31712578 PMC6848502

[91] Cromie GA, Hyppa RW, Smith GR. The fission yeast BLM homolog Rqh1 promotes meiotic recombination. Genetics. 2008;179:1157–67. 10.1534/genetics.108.088955. 18562672 PMC2475723

[92] Hyppa RW, Cho JD, Nambiar M et al. Redirecting meiotic DNA break hotspot determinant proteins alters localized spatial control of DNA break formation and repair. Nucleic Acids Res. 2022;50:899–914. 10.1093/nar/gkab1253. 34967417 PMC8789058

[93] Latypov V, Rothenberg M, Lorenz A et al. Roles of Hop1 and Mek1 in meiotic chromosome pairing and recombination partner choice in *Schizosaccharomyces pombe*. Mol Cell Biol. 2010;30:1570–81. 10.1128/MCB.00919-09. 20123974 PMC2838064

[94] Brown SD, Jarosinska OD, Lorenz A. Genetic interactions between the chromosome axis-associated protein Hop1 and homologous recombination determinants in *Schizosaccharomyces pombe*. Curr Genet. 2018;64:1089–104. 10.1007/s00294-018-0827-7. 29550859 PMC6153652

[95] Kim ST, Lim DS, Canman CE et al. Substrate specificities and identification of putative substrates of ATM kinase family members. J Biol Chem. 1999;274:37538–43. 10.1074/jbc.274.53.37538. 10608806

[96] Baumann P, Cech TR. Protection of telomeres by the Ku protein in fission yeast. Mol Biol Cell. 2000;11:3265–75. 11029034 10.1091/mbc.11.10.3265 PMC14990

[97] Manolis KG, Nimmo ER, Hartsuiker E et al. Novel functional requirements for non-homologous DNA end joining in *Schizosaccharomyces pombe*. EMBO J. 2001;20:210–21. 11226171 10.1093/emboj/20.1.210 PMC140209

[98] Usui T, Ogawa H, Petrini JH. A DNA damage response pathway controlled by Tel1 and the Mre11 complex. Mol Cell. 2001;7:1255–66. 11430828 10.1016/s1097-2765(01)00270-2

[99] Morales M, Theunissen JW, Kim CF et al. The Rad50S allele promotes ATM-dependent DNA damage responses and suppresses ATM deficiency: implications for the Mre11 complex as a DNA damage sensor. Genes Dev. 2005;19:3043–54. 16357220 10.1101/gad.1373705 PMC1315407

[100] Wilson S, Tavassoli M, Watts FZ. *Schizosaccharomyces pombe* Rad32 protein: a phosphoprotein with an essential phosphoesterase motif required for repair of DNA double strand breaks. Nucleic Acids Res. 1998;26:5261–9. 9826747 10.1093/nar/26.23.5261 PMC147988

[101] Chuang YC, Smith GR. Dynamic configurations of meiotic DNA-break hotspot determinant proteins. J Cell Sci. 2022;135:jcs259061. 10.1242/jcs.259061. 35028663 PMC8918816

[102] Hyppa RW, Fowler KR, Cipak L et al. DNA intermediates of meiotic recombination in synchronous *S. pombe* at optimal temperature. Nucleic Acids Res. 2013;42:359–69. 10.1093/nar/gkt861. 24089141 PMC3874177

[103] Brown SD, Audoynaud C, Lorenz A. Intragenic meiotic recombination in *Schizosaccharomyces pombe* is sensitive to environmental temperature changes. Chromosome Res. 2020;28:195–207. 10.1007/s10577-020-09632-3. 32303869 PMC7242256

[104] Pryce DW, Lorenz A, Smirnova JB et al. Differential activation of *M26*-containing meiotic recombination hot spots in *Schizosaccharomyces pombe*. Genetics. 2005;170:95–106. 10.1534/genetics.104.036301. 15744055 PMC1449712

[105] Williams RS, Williams JS, Tainer JA. Mre11-Rad50-Nbs1 is a keystone complex connecting DNA repair machinery, double-strand break signaling, and the chromatin template. Biochem Cell Biol. 2007;85:509–20. 10.1139/O07-069. 17713585

[106] Hartsuiker E, Mizuno K, Molnar M et al. Ctp1^CtIP^ and the Rad32^Mre11^ nuclease activity are required for Rec12^Spo11^ removal but Rec12^Spo11^ removal is dispensable for other MRN-dependent meiotic functions. Mol Cell Biol. 2009;29:1671–81. 10.1128/MCB.01182-08. 19139281 PMC2655602

[107] Limbo O, Porter-Goff ME, Rhind N et al. Mre11 nuclease activity and Ctp1 regulate Chk1 activation by Rad3^ATR^ and Tel1^ATM^ checkpoint kinases at double-strand breaks. Mol Cell Biol. 2011;31:573–83. 10.1128/MCB.00994-10. 21098122 PMC3028622

[108] You Z, Chahwan C, Bailis J et al. ATM activation and its recruitment to damaged DNA require binding to the C terminus of Nbs1. Mol Cell Biol. 2005;25:5363–79. 10.1128/MCB.25.13.5363-5379.2005. 15964794 PMC1156989

[109] Limbo O, Yamada Y, Russell P. Mre11-Rad50-dependent activity of ATM/Tel1 at DNA breaks and telomeres in the absence of Nbs1. MBoC. 2018;29:1389–99. 10.1091/mbc.E17-07-0470. 29851556 PMC5994899

[110] Lopez Ruiz LM, Johnson D, Gittens WH et al. Meiotic prophase length modulates Tel1-dependent DNA double-strand break interference. PLoS Genet. 2024;20:e1011140. 10.1371/journal.pgen.1011140. 38427688 PMC10936813

[111] Wang MF, Li MY, Yang YC et al. Mug20-Rec25-Rec27 binds DNA and enhances meiotic DNA break formation via phase-separated condensates. Nucleic Acids Res. 2025;53:gkaf123. 40037704 10.1093/nar/gkaf123 PMC11879393

[112] Rog O, Kohler S, Dernburg AF. The synaptonemal complex has liquid crystalline properties and spatially regulates meiotic recombination factors. eLife. 2017;6:e21455. 10.7554/eLife.21455. 28045371 PMC5268736

[113] Claeys Bouuaert C, Pu S, Wang J et al. DNA-driven condensation assembles the meiotic DNA break machinery. Nature. 2021;592:144–9. 10.1038/s41586-021-03374-w. 33731927 PMC8016751

[114] de Jager M, van Noort J, van Gent DC et al. Human Rad50/Mre11 is a flexible complex that can tether DNA ends. Mol Cell. 2001;8:1129–35. 10.1016/S1097-2765(01)00381-1. 11741547

[115] Williams RS, Moncalian G, Williams JS et al. Mre11 dimers coordinate DNA end bridging and nuclease processing in double-strand-break repair. Cell. 2008;135:97–109. 10.1016/j.cell.2008.08.017. 18854158 PMC2681233

[116] Cassani C, Gobbini E, Wang W et al. Tel1 and Rif2 regulate MRX functions in end-tethering and repair of DNA double-strand breaks. PLoS Biol. 2016;14:e1002387. 10.1371/journal.pbio.1002387. 26901759 PMC4762649

[117] Sakuno T, Tashiro S, Tanizawa H et al. Rec8 Cohesin-mediated Axis-loop chromatin architecture is required for meiotic recombination. Nucleic Acids Res. 2022;50:3799–816. 10.1093/nar/gkac183. 35333350 PMC9023276

[118] Kar FM, Vogel C, Hochwagen A. Meiotic DNA breaks activate a streamlined phospho-signaling response that largely avoids protein-level changes. Life Sci Alliance. 2022;5:e202201454. 10.26508/lsa.202201454 . 36271494 PMC9438802

[119] Guo H, Stamper EL, Sato-Carlton A et al. Phosphoregulation of DSB-1 mediates control of meiotic double-strand break activity. eLife. 2022;11:e77956. 10.7554/eLife.77956. 35758641 PMC9278955

