# Supplementary material for: Mutual, spatially limited control of meiotic DNA break formation by Mre11–Rad50–Nbs1 DNA repair complex and Tel1 (ATM) protein kinase
Source: Nucleic Acids Res. 2025 Dec 22;53(22):gkaf1405. doi: 10.1093/nar/gkaf1405 (PMC12721321; doi:10.1093/nar/gkaf1405)
Supplement: gkaf1405_Supplemental_File [file gkaf1405_supplemental_file.pdf]

Mutual, Spatially Limited Control of Meiotic DNA Break Formation by Mre11-Rad50-Nbs1 DNA Repair Complex and Tel1 (ATM) Protein Kinase

Randy W. Hyppa and Gerald R. Smith

Supplemental Information

Table S1. ***S. pombe*** **strains**

Strain	Genotype	Used in
GP887	*h+ ade6-M26 ura1-171*	Table 1
GR76	*h- ade6-3049 ura1::hphMX6 pat1-as* (L95G)-*kanMX6*	Table 1
GP7080	*h- ura4-D18*	strain construction
GP9400	*h- tel1::ura4*$^{+}$ *ura4-D18*	strain construction
GP9402	*h- tel1-kd ura4-D18*	strain construction
GP9901	*h- ade6-3049 pat1-as* (L95G)-*kanMX6 rad50S*	Figure 1, 2
GR15	*h- ade6-3049 tel1::natMX6 pat1-as* (L95G)-*kanMX6 ura4-aim rad50S*	Figure 1, 2
GR19	*h- ade6-3049 tel1::natMX6 rec10-144 pat1-as* (L95G)-*kanMX6 ura4-aim rad50S*	Figure 1, 2
GR22	*h- ade6-3049 rec10-144 pat1-as* (L95G)-*kanMX6 ura4-aim rad50S*	Figure 1, 2
GR83	*h- ura1::hphMX6 pat1-as* (L95G)-*kanMX6 rad50S*	Figure 1, 2, 3
GR107	*h- ade6-M26 rec11::kanMX6 ura1::hphMX6*	
GR108	*h- ade6-M26 rec11::kanMX6 ura1::hphMX6 pat1-as* (L95G)-*kanMX6 rad50S*	Figure 1, 2, 3, 6, S7
GR109	*h- ade6-M26 rec11::kanMX6 tel1::natMX6 ura1::hphMX6 pat1-as* (L95G)-*kanMX6*	Table 1
GR110	*h- ade6-M26 rec11::kanMX6 tel1::natMX6 ura1::hphMX6 pat1-as* (L95G)-*kanMX6 rad50S*	Figure 1, 2, 3
GR112	*h+ ade6-52 rec11::kanMX6 ura1-171*	Table 1
GR113	*h- ade6-52 rec11::kanMX6 tel1::natMX6 ura1-171*	Table 1
GR114	*h+ ade6-52 rec11::kanMX6 tel1::natMX6 ura1-171*	Table 1
GR115	*h- ade6-M26 rec10-144 pat1-as* (L95G)-*kanMX6 ura1::hphMX6*	Table 1
GR116	*h- ade6-M26 rec10-144 pat1-as* (L95G)-*kanMX6 ura1::hphMX6 rad50S*	Figure 1, 2
GR117	*h- ade6-M26 tel1::natMX6 rec10-144 pat1-as* (L95G)-*kanMX6 ura1::hphMX6*	Table 1
GR118	*h- ade6-M26 tel1::natMX6 rec10-144 pat1-as* (L95G)-*kanMX6 ura1::hphMX6 rad50S*	Figure 1, 2, 3

GR119	*h- ade6-M26 tel1::natMX6 pat1-as* (L95G)-*kanMX6 ura1::hphMX6 rad50S*	Figure 1, 2, 3
GR120	*h- ade6-M26 tel1::natMX6 pat1-as* (L95G)-*kanMX6 ura1::hphMX6*	Table 1
GR122	*h- ade6-52 rec10-144 ura1-171*	Table 1
GR123	*h- ade6-52 tel1::natMX6 rec10-144 ura1-171*	Table 1
GR124	*h+ ade6-52 tel1::natMX6 ura1-171*	Table 1
GR125	*h+ ade6-52 tel1::natMX6 rec10-144 ura1-171*	Table 1
GR142	*h- ade6-52 rec10-144 ura1::hphMX6 mbs1-20 pat1-as* (L95G)-*kanMX6*	Table 1
GR144	*h+ ade6-52 tel1::natMX6 rec10-144 ura1::hphMX6 mbs1-20 pat1-as* (L95G)-*kanMX6*	Table 1
GR145	*h+ ade6-52 tel1::natMX6 ura1::hphMX6 mbs1-20 pat1-as* (L95G)-*kanMX6*	Table 1
GR146	*h- ade6-52 ura1::hphMX6 mbs1-20 pat1-as* (L95G)-*kanMX6*	Table 1
GR185	*h- ade6-M26 rec11::kanMX6 pat1-as* (L95G)-*kanMX6 rad50S*	Figure 1, 2, S7
GR186	*h- ade6-M26 rec11::kanMX6 rec10-144 ura1::hphMX6 pat1-as* (L95G)-*kanMX6 rad50S*	Table 1
GR190	*h- ade6-M26 rec11::kanMX6 tel1::natMX6 rec10-144 ura1::hphMX6*	Table 1
GR191	*h- ade6-M26 rec11::kanMX6 tel1::natMX6 rec10-144 ura1::hphMX6 pat1-as* (L95G)-*kanMX6 rad50S*	Figure S2, Table 1
GR193	*h- ade6-M26 rec11::kanMX6 tel1::natMX6 pat1-as* (L95G)-*kanMX6 rad50S*	Figure 1, 2
GR194	*h+ ade6-52 rec11::kanMX6 rec10-144 ura1-171*	Table 1
GR195	*h+ ade6-52 rec11::kanMX6 tel1::natMX6 rec10-144 ura1-171*	Table 1
GR230	*h- ade6-3049 rec27-184::kanMX6 pat1-as* (L95G)-*kanMX6 rad50S*	Figure 1,2
GR412	*h- ade6-52 rec27-184::kanMX6 ura1::hphMX6 pat1-as* (L95G)-*kanMX6 rad50S*	Figure 1, 2, 3
GR414	*h+ ade6-M26 rec11::kanMX6 tel1::natMX6 rec27-184::kanMX6 ura1::hphMX6 pat1-as* (L95G)-*kanMX6 rad50S*	Figure S2
GR417	*h+ ade6-M26 tel1::natMX6 rec27-184::kanMX6 ura1::hphMX6 pat1-as* (L95G)-*kanMX6 rad50S*	Figure 1, 2, 3
GR507	*h- ade6-M26 tel1::natMX6 rec12-164* (Y98F) *pat1-as* (L95G)-*kanMX6 ura1::hphMX6 rad50S*	Figure S1
GR508	*h- ade6-M26 rec12-164* (Y98F) *pat1-as* (L95G)-*kanMX6 ura1::hphMX6 rad50S*	Figure S1
GR514	*h+ ade6-M26 tel1::natMX6 mug20::natMX6 pat1-as* (L95G)-*kanMX6 ura1::hphMX6 rad50S*	Figure S1, S2
GR515	*h+ ade6-52 mug20::natMX6 pat1-as* (L95G)-*kanMX6 ura1::hphMX6 rad50S*	Figure S1, S2

GR525	*h+ ade6-M26 tel1::natMX6 rec8-206::kanMX6 pat1-as* (L95G)-*kanMX6 ura1::hphMX6 rad50S*	Figure S1, S2
GR526	*h+ ade6-M26 rec8-206::kanMX6 pat1-as* (L95G)-*kanMX6 ura1::hphMX6 rad50S*	Figure S1, S2
GR527	*h+ ade6-M26 rec25-180::kanMX6 pat1-as* (L95G)-*kanMX6 ura1::hphMX6 rad50S*	Figure S1, S2
GR528	*h+ ade6-M26 tel1::natMX6 rec25-180::kanMX6 pat1-as* (L95G)-*kanMX6 ura1::hphMX6 rad50S*	Figure S1, S2
GR561	*h+ ade6-52 hop1::ura4$^+$ pat1-as* (L95G)-*kanMX6 ura1::hphMX6 rad50S*	Figure 4
GR563	*h+ ade6-M26 tel1::natMX6 hop1::ura4$^+$ pat1-as* (L95G)-*kanMX6 ura1::hphMX6 rad50S*	Figure 4
GR565	*h- ade6-M26 rec11::kanMX6 hop1::ura4$^+$ pat1-as* (L95G)-*natMX6 ura1::hphMX6 rad50S ura4-D18*	strain construction
GR567	*h+ ade6-M26 rec11::kanMX6 hop1::ura4$^+$ pat1-as* (L95G)-*natMX6 ura1::hphMX6 rad50S*	Figure 4
GR570	*h- ade6-M26 rec11::kanMX6 hop1-325-5A pat1-as* (L95G)-*natMX6 ura1::hphMX6 rad50S ura4-D18*	strain construction
GR622	*h+ ade6-52 nbs1::kanMX6 pat1-as* (L95G)-*kanMX6 ura1::hphMX6*	Figure 5, S5
GR624	*h- ade6-M26 rec11::kanMX6 nbs1::kanMX6 pat1-as* (L95G)-*kanMX6 ura1::hphMX6*	Figure 6, S7
GR625	*h- ade6-M26 rec11::kanMX6 nbs1::kanMX6 pat1-as* (L95G)-*kanMX6 ura1::hphMX6 rad50S*	Figure 6, S7
GP626	*h+ ade6-52 mre11::ura4$^+$ pat1-as* (L95G)-*kanMX6 ura1::hphMX6 ura4-D18*	Figure 5, S5, S8
GR627	*h+ ade6-M26 rec11::kanMX6 mre11::ura4$^+$ pat1-as* (L95G)-*kanMX6 ura1::hphMX6 ura4-D18*	Figure 6, S7, S8
GP685	*h+ mre11-D65N pat1-as* (L95G)-*kanMX6 ura1::hphMX6*	Figure 5, S5, S8
GR686	*h- ade6-M26 rec11::kanMX6 mre11-D65N pat1-as* (L95G)-*kanMX6 ura1::hphMX6 ura4-D18*	Figure 6, S7, S8
GR687	*h- ctp1::kanMX6 pat1-as* (L95G)-*kanMX6 ura1::hphMX6 ura4-D18*	Figure 5, S5
GR689	*h+ ade6-52 rad50::kanMX6 pat1-as* (L95G)-*kanMX6 ura1::hphMX6*	Figure 5, S5
GR690	*h+ ade6-M26 rec11::kanMX6 rad50::kanMX6 pat1-as* (L95G)-*kanMX6 ura1::hphMX6*	Figure 6, S7
GR692	*h+ ade6-M26 tel1::natMX6 mre11::ura4$^+$ pat1-as* (L95G)-*kanMX6 ura1::hphMX6 ura4-D18*	Figure 5, S5
GR693	*h+ ade6-M26 rec11::kanMX6 tel1-280-kd ura1::hphMX6 pat1-as* (L95G)-*kanMX6 rad50S*	Figure 6, S7, S8
GR694	*h+ ade6-M26 tel1-280-kd ura1::hphMX6 pat1-as* (L95G)-*kanMX6 rad50S*	Figure 5, S5, S8
GR702	*h+ ade6-52 pku70::ura4$^+$ pat1-as* (L95G)-*kanMX6 ura1::hphMX6 rad50S*	Figure S6

GR705	*h- ade6-M26 tel1::kanMX6 pku70::ura4⁺ pat1-as* (L95G)-*kanMX6 ura1::hphMX6 rad50S*	Figure S6
GR706	*h- ade6-52 mre11::ura4⁺ pku70::ura4⁺ pat1-as* (L95G)-*kanMX6 ura1::hphMX6*	Figure S6
GR732	*h+ ade6-M26 tel1::natMX6 mre11::ura4⁺ pku70::ura4⁺ pat1-as* (L95G)-*kanMX6 ura1::hphMX6*	Figure S6
GR733	*h+ ade6-M26 tel1::natMx6 rec27-184::kanMX6 pat1-as* (L95G)-*kanMX6 rad50S*	Figure 1, 2
GR736	*h+ ade6-52 rec11::kanMX6 tel1::natMx6 hop1::ura4⁺ pat1-as* (L95G)-*kanMX6 ura1::hphMX6 rad50S*	Figure 4
GR846	*h+ ade6-M26 tel1::natMx6 mre11-D65N pat1-as* (L95G)-*natMX6 ura1::hphMX6*	Figure S3, S4
GR847	*h+ ade6-M26 rec11::kanMX6 tel1::natMx6 mre11-D65N pat1-as* (L95G)-*natMX6 ura1::hphMX6*	Figure S3, S4
GR848	*h+ ade6-M26 rec27::kanMX6 tel1::natMx6 mre11-D65N pat1-as* (L95G)-*natMX6 ura1::hphMX6*	Figure S3, S4
GR849	*h+ ade6-M26 rec27::kanMX6 mre11-D65N pat1-as* (L95G)-*natMX6 ura1::hphMX6*	Figure S3, S4
GR851	*h+ hop1::kanMx6 mre11-D65N pat1-as* (L95G)-*natMX6 ura1::hphMX6*	Figure S3, S4
GR853	*h+ hop1-325-5A pat1-as* (L95G)-nat*MX6 ura1::hphMX6 rad50S*	Figure S3, S4

Strains were constructed by standard matings (Smith, 2009) or as described in Materials and Methods. Genealogies are available upon request. Alleles used in this study were from the following sources: *ura1::hphMX6* (1), *pat1-as* (L95G)-*kanMX6* (2), *rad50S* (3), *ura4⁺-aim* (4), *tel1::kanMX6* (5), *rec10-144* (6), *rec11::kanMX6* (7), *mbs1-20* (5), *rec27-184::kanMX6* (8), *rec12-164* (Y98F) (9), *mug20::natMX6* (10), *rec8::kanMX6* (11), *rec25-180::kanMX6* (8), *nbs1::kanMX6* (12), *mre11::ura4⁺* (13), *mre11-D65N* (13), *ctp1::kanMX6* (14), *rad50::kanMX6* (3), *tel1-280-kd* (this study), *pku70::ura4⁺* (15), *hop1::ura4⁺* (Nojima Lab), *hop1::kanMX6* (16), *hop1-5A* (this study).

Table S2. Oligonucleotides

Name	Nucleotide sequence (5’ → 3’)	Used to construct
OL757	GCACCATTTCCAAAGACCCATACAG	DNA probe, Figure 2
OL758	ATCAAAGAGCGAACACATCTCCATT	DNA probe, Figure 2
OL1041	CGATAGGTTTCGTCGTACCC	DNA probe, Figures 3A, 4B, 5A, 6A, S2A, S4A, S6B, S8A
OL1042	CCGCTTCCCTGATAATGACTGAC	DNA probe, Figures 3A, 4B, 5A, 6A, S2A, S4A, S6B, S8A
OL5252	GCTTATGCATAACGATTGCG	DNA probe, Figures 1, 4A, S1, S3, S5, S6A, S7
OL5253	CTATCTCTACTGTTTCCACC	DNA probe, Figures 1, 4A, S1, S3, S5, S6A, S7
OL5254	TCGAGAGTTACGCTGATCCAGTGCAATGGTTTA CAACGCAAACAAATTACGCACGAAGCACTGCTG TTGCTTCTGTCCTGAGCTTGTGATATTGACGAAA	*tel1::ura4*$^{+}$ mutation
OL5255	ACCCCGGTAATTCCCATACCATCTACAACGTCTC TTGTAAGTCGAAAGGGAACACATTCAGGAACGG GTAGTTTTTTACCAGCTTAGCTACAAATCCCAC	*tel1::ura4*$^{+}$ mutation
OL5256	TGGTCTTTGAGTACTTGTCG	*tel1-kd* mutation transformation
OL5257	TCTCAAAGGTGAAATAAGCC	*tel1-kd* mutation transformation

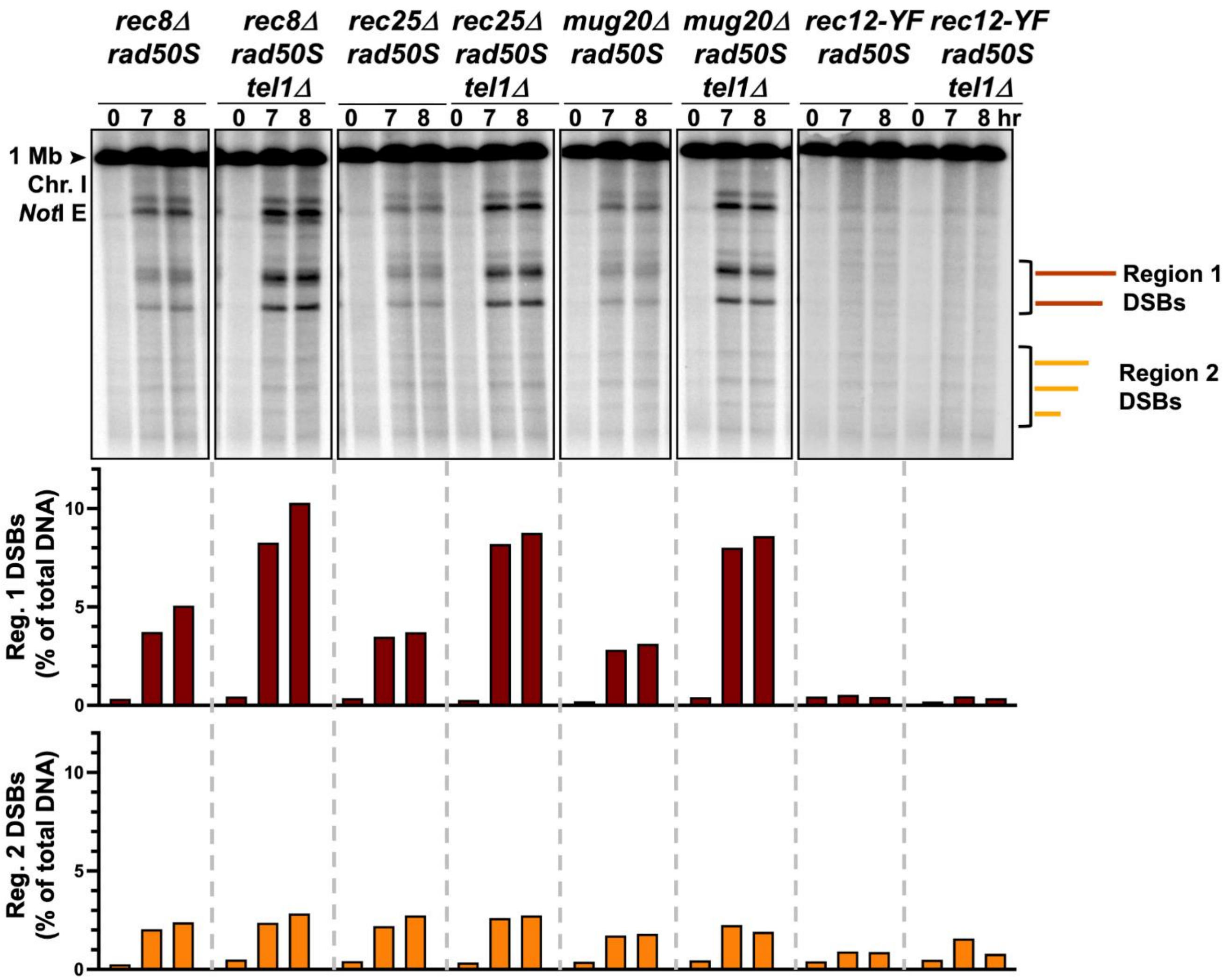

Figure S1. Mutants of additional meiosis-specific components of cohesin and LinE complexes have similar DSB phenotypes

DSBs were measured in meiotic cohesin mutant *rec8*Δ, LinE complex mutants *rec25*Δ and *mug20*Δ, and a catalytically dead mutant of the DSB-forming protein Rec12 [(*rec12-164* (Y98F)], in both *tel1*⁺ and *tel1*Δ strains. *Not*I-digested DNA was Southern blot hybridized with a [^{32}P]-labelled DNA probe on the left end of the 1 Mb *Not*I E fragment on Chromosome I (see Figure 1), and DSBs were measured across an approximately 0.5 Mb interval. DSB frequencies across the interval (as % of total DNA) are represented in the graphs beneath the Southern blot images: upper graph, Region 1; lower graph, Region 2. For each strain, the DSB frequencies are from one experiment.

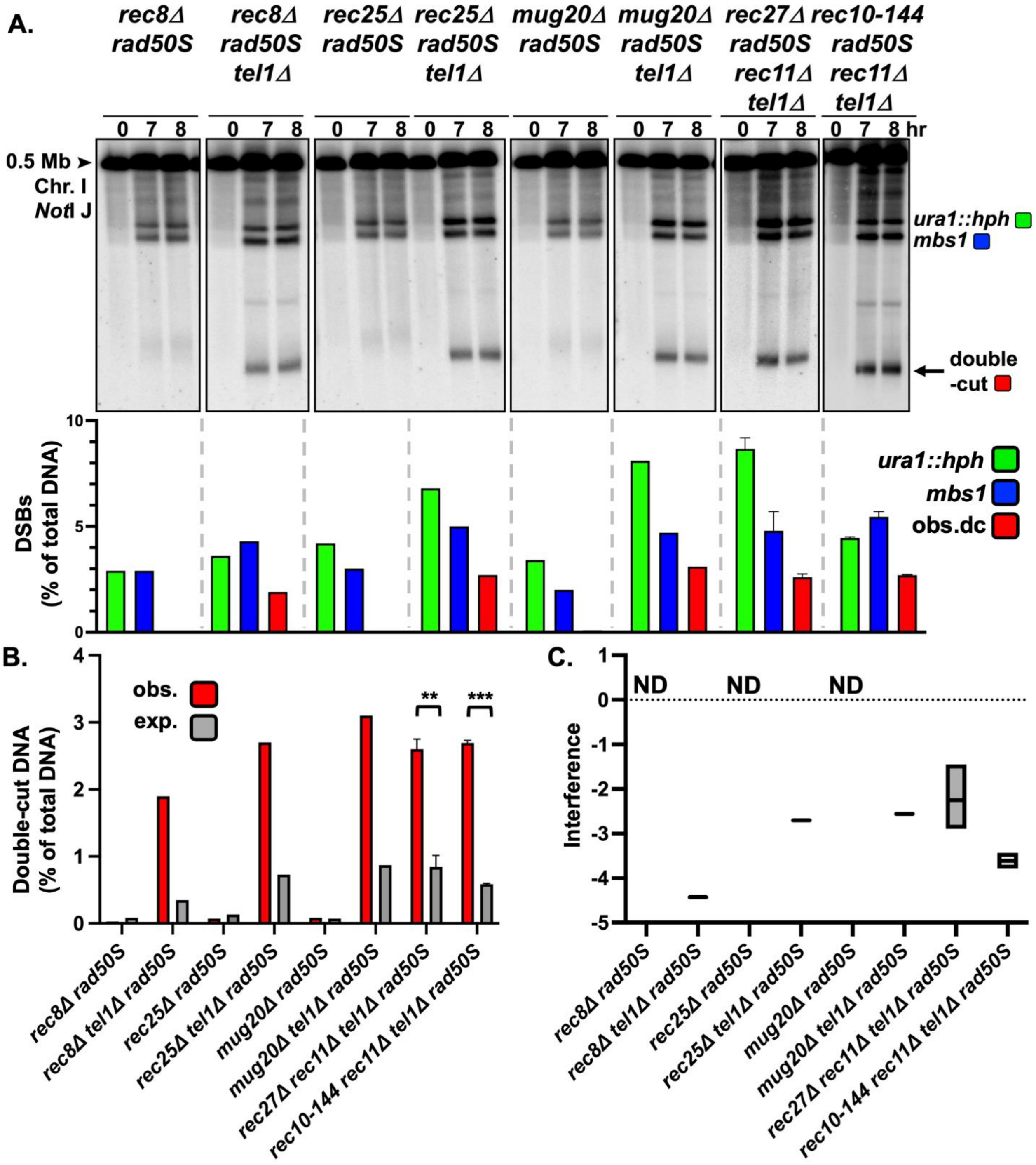

Figure S2. Additional meiosis-specific mutants of the cohesin and LinE complexes have strongly negative DSB interference

(A) The frequency of double-cut DNA was determined in meiotic cohesin mutant *rec8*Δ, LinE complex mutants *rec25*Δ and *mug20*Δ, and double complex mutants *rec27*Δ *rec11*Δ and *rec27*Δ

*rec11*Δ, each with *tel1*$^{+}$ or *tel1Δ. Not*I-digested DNA was Southern blot hybridized with a [^{32}P]-labelled DNA probe located in the middle of the 0.5 Mb *Not*I J fragment on Chromosome I, between the inserted DSB hotspot *ura1::hph* and *mbs1,* to detect DNA chromatids that were cut at both DSB hotspots. The mean DSB frequencies were from the 7- and 8-hour timepoints of *ura1::hph* DSBs (green), *mbs1* DSBs (blue), and observed DNA double-cut at both *ura1::hph* and *mbs1* (red) are shown on the graph (as % of total DNA). (B) The double-cut frequencies are very low and the observed (red) and expected (% *ura1*::hph x *% mbs1*; gray) double-cut frequencies (as % of total DNA) are plotted on a separate graph for visibility. Observed and expected values are the mean of the 7- and 8-hour timepoints. The values on the bar graph without error bars are from single data points; otherwise, the mean and SEM are shown from 3 experiments. (C) DSB interference, calculated as 1 – (observed double-cut DNA/expected double-cut DNA), for each strain is plotted on the graph; line indicates the calculated value for single data points; boxes indicate the minimum and maximum. Interference was not determined (ND) for strains in which the expected double-cut DNA was <0.1%.

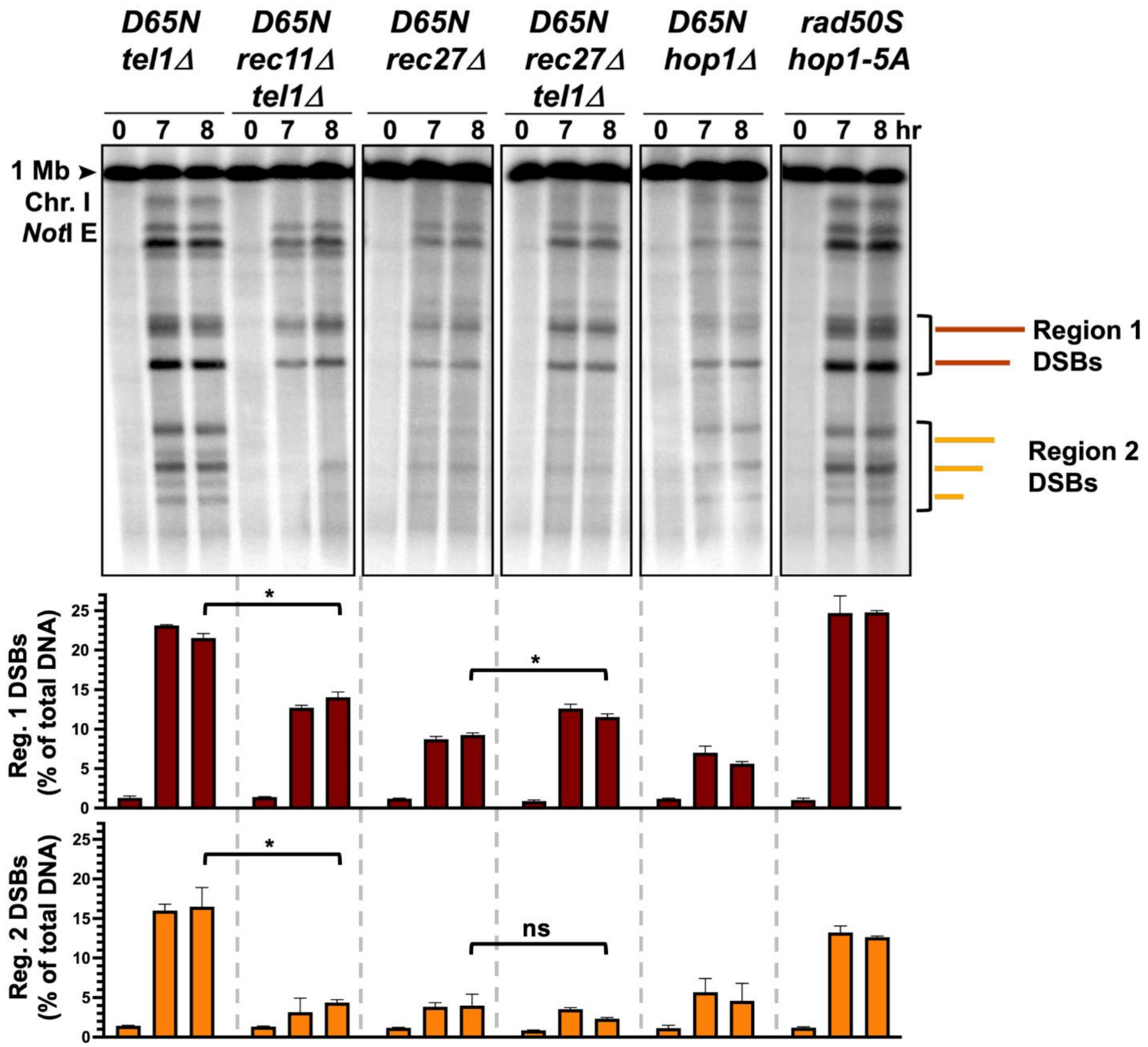

Figure S3. DSBs assayed in *mre11-D65N* are similar to those in *rad50S*

DSBs were measured in mutants of the MRN DNA repair complex, and a *rad50S tel1-kd* strain for comparison (see Figure 1 for *rad50S*). *Not*I-digested DNA was Southern blot-hybridized with a [^{32}P]-labelled DNA probe on the left end of the 1 Mb *Not*I E fragment on Chromosome I (see Figure 1), and DSBs were measured across an approximately 0.5 Mb (designated Regions 1 and 2). DSB frequencies (as % of total DNA) are represented in the graphs beneath the Southern blot images: upper graph, Region 1; lower graph, Region 2. For each strain, the mean and SEM are shown from 3 or 4 experiments.

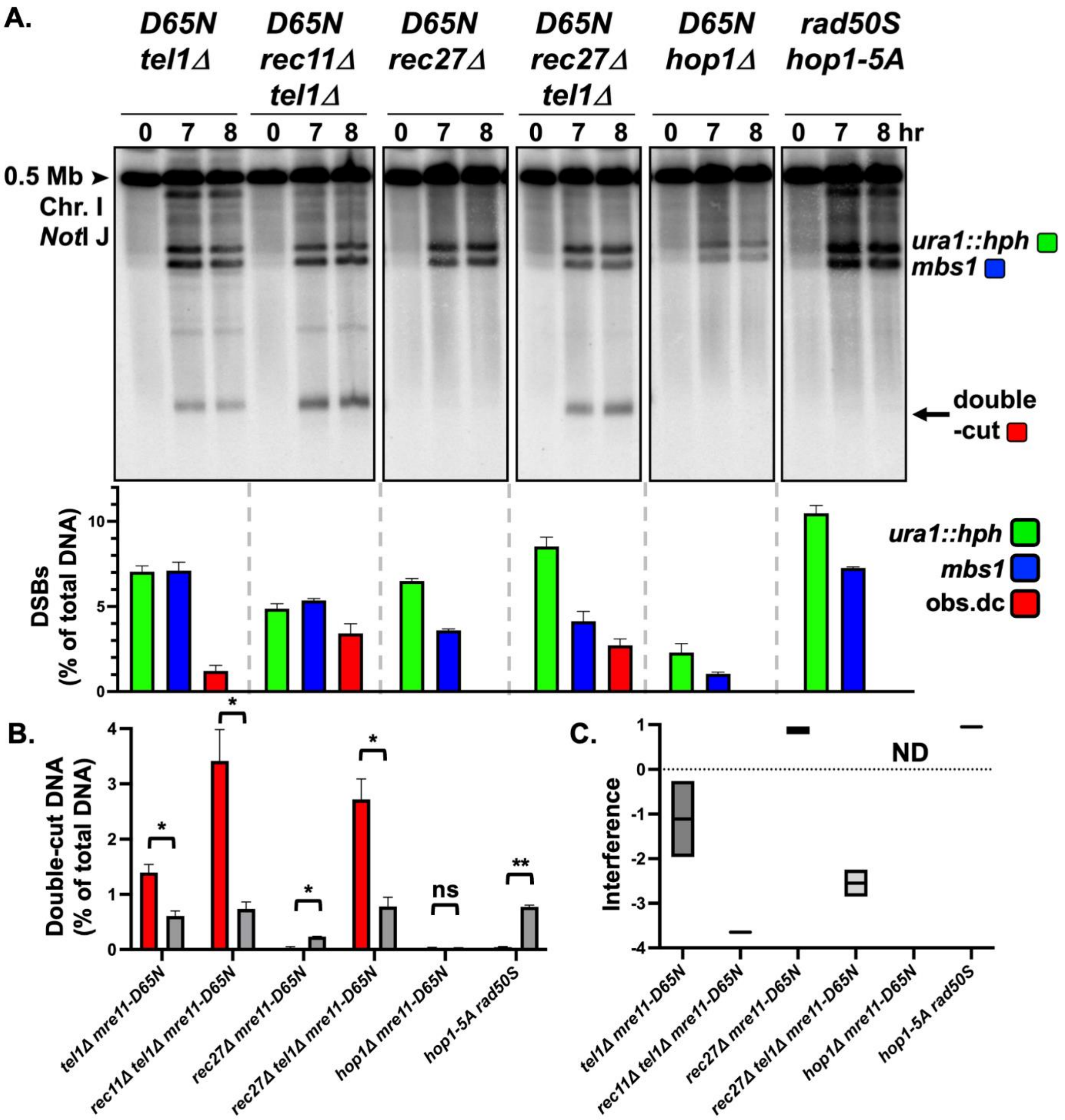

Figure S4. DSB interference is positive in *mre11-D65N* and *hop1-5A* phosphorylation negative mutants and negative in *tel1Δ.*

A) The frequency of double-cut DNA was determined in meiotic cohesin mutant *rec8*Δ, LinE complex mutants *rec25*Δ and *mug20*Δ, and double complex mutants *rec27*Δ *rec11*Δ and *rec27*Δ *rec11*Δ, each with *tel1*$^{+}$ or *tel1Δ. Not*I-digested DNA was Southern blot hybridized with a [^{32}P]-labelled DNA probe located in the middle of the 0.5 Mb *Not*I J fragment on Chromsome I, between the inserted DSB hotspot *ura1::hph* and *mbs1* to detect DNA chromatids that were cut

at both DSB hotspots. The mean DSB frequencies were from the 7- and 8-hour timepoints of *ura1::hph* DSBs (green), *mbs1* DSBs (blue), and observed DNA double-cut at both *ura1::hph* and *mbs1* (red) are shown on the graph (as % of total DNA). (B) The double-cut frequencies are very low and the observed (red) and expected (% *ura1*::hph x *% mbs1*; gray) double-cut frequencies (as % of total DNA) are plotted on a separate graph for visibility. Observed and expected values are the mean of the 7- and 8-hour timepoints. The values on the bar graph without error bars are from single data points; otherwise, the mean and SEM are shown from 3 experiments. (C) DSB interference, calculated as 1 – (observed double-cut DNA/expected double-cut DNA), for each strain is plotted on the graph; line indicates the calculated value for single data points; boxes indicate the minimum and maximum. Interference was not determined (ND) for strains in which the expected double-cut DNA was <0.1%.

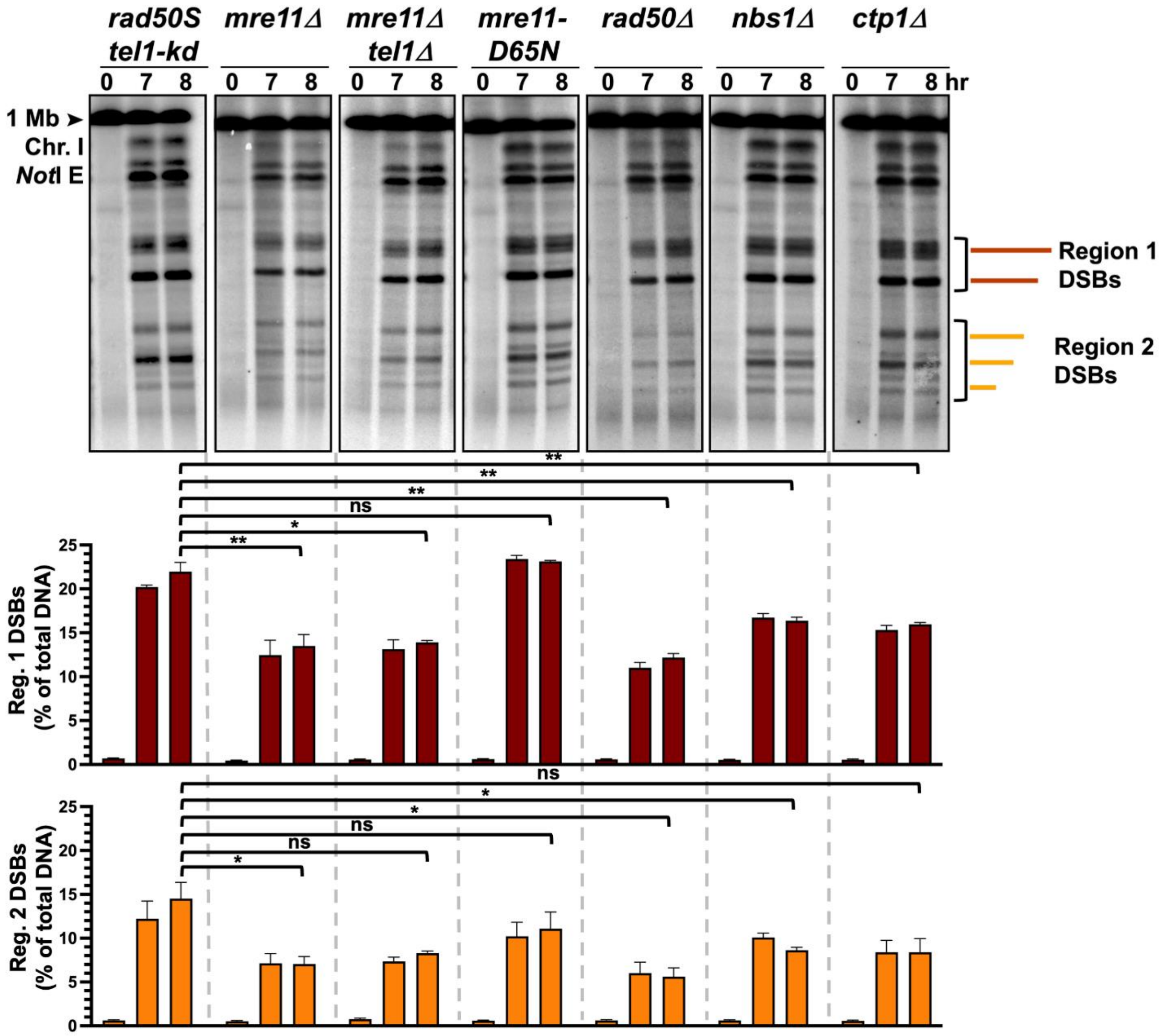

Figure S5. DSBs are formed at slightly reduced frequency in MRN null mutants

DSBs were measured in mutants of the MRN DNA repair complex, and a *rad50S tel1-kd* strain for comparison (see Figure 1 for *rad50S*). *Not*I-digested DNA was Southern blot-hybridized with a [^{32}P]-labelled DNA probe on the left end of the 1 Mb *Not*I E fragment on Chromosome I (see Figure 1), and DSBs were measured across an approximately 0.5 Mb (designated Regions 1 and 2). DSB frequencies (as % of total DNA) are represented in the graphs beneath the Southern blot images: upper graph, Region 1; lower graph, Region 2. For each strain, the mean and SEM are shown from 3 or 4 experiments.

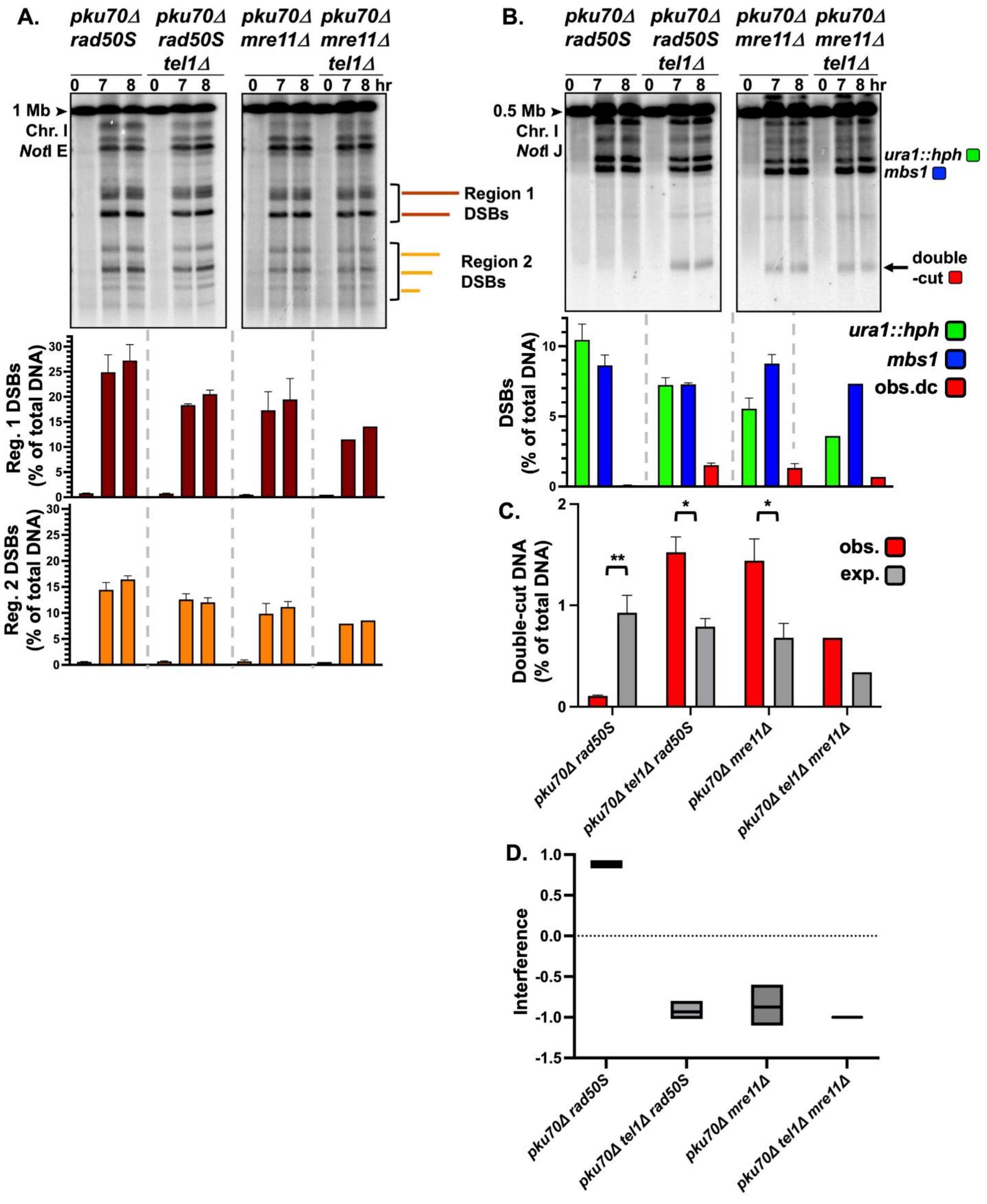

Figure S6. ***pku70*** **Δ has no observable effect on DSB formation or interference**

(A) DSBs were measured in the NHEJ mutant *pku70*Δ, with and without Mre11 and Tel1. *Not*I-digested DNA was Southern blot-hybridized with a [^{32}P]-labelled DNA probe on the left end of the 1 Mb *Not*I E fragment on Chromosome I (see Figure 1), and DSBs were measured across

an approximately 0.5 Mb (designated Regions 1 and 2). DSB frequencies (as % of total DNA) are represented in the graphs beneath the Southern blot images: upper graph, Region 1; lower graph, Region 2 (see Figure 1 to compare *pku70*$^{+}$). For each strain, the mean and range are shown from two experiments (one for *pku70*Δ *tel1*Δ *mre11*Δ). (B) The frequency of double-cut DNA was measured in *pku70*Δ, with and without Mre11 and Tel1. *Not*I-digested DNA was Southern blot hybridized with a [^{32}P]-labelled DNA probe located in the middle of the 0.5 Mb *Not*I J fragment on Chromosome 1, between the inserted DSB hotspot *ura1::hph* and *mbs1* to detect DNA chromatids that were cut at both DSB hotspots. The mean DSB frequencies from the 7- and 8-hour timepoints of *ura1::hph* DSBs (green), *mbs1* DSBs (blue), and observed DNA double-cut at both *ura1::hph* and *mbs1* (red) are shown on the graph (as % of total DNA).. (C) The double-cut frequencies are very low and the observed (red) and expected (%*ura1::hph* x % *mbs1*, gray) double-cut frequencies (as % of total DNA) are plotted on a separate graph for visibility. Observed and expected values are the mean of the 7- and 8-hour timepoints. For each strain, the mean and range are shown from two experiments (one for *pku70D tel1D mre11*). (D) DSB interference, calculated as 1 – (observed double-cut DNA/expected double-cut DNA), for each strain is plotted on the graph; line in each box indicates the median; the minimum and maximum values measured are at the bottom and top of each box.

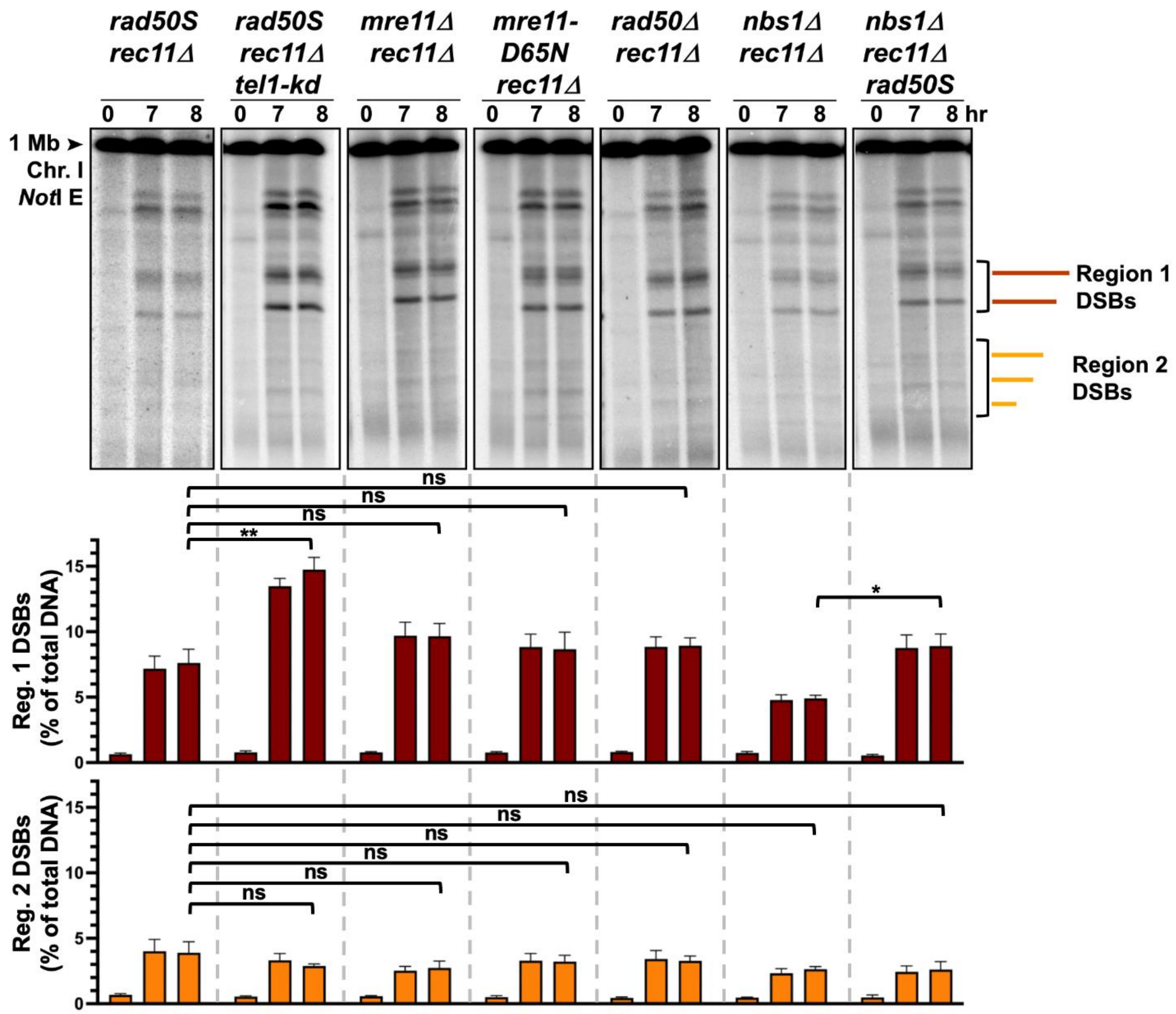

Figure S7. *mre11*Δ and *rad50*Δ, like *tel1*Δ, modestly increase DSB formation in *rec11*Δ cohesin mutants

DSBs were measured in the cohesin mutant *rec11*Δ with additional mutations of the MRN DNA repair complex, and *tel1-kd* for comparison. *Not*I-digested DNA was Southern blot hybridized with a [^{32}P]-labelled DNA probe on the left end of the 1 Mb *Not*I E fragment (see Figure 1), and DSBs were measured across two sections of an approximately 0.5 Mb on Chromosome I (designated Regions 1 and 2). DSB frequencies (as % of total DNA) are represented in the graphs beneath the Southern blot images: upper graph, Region 1; lower graph, Region 2. For each strain, the mean and SEM are shown from 3 to 6 experiments. *rad50S rec11Δ* data are from Figure 1.

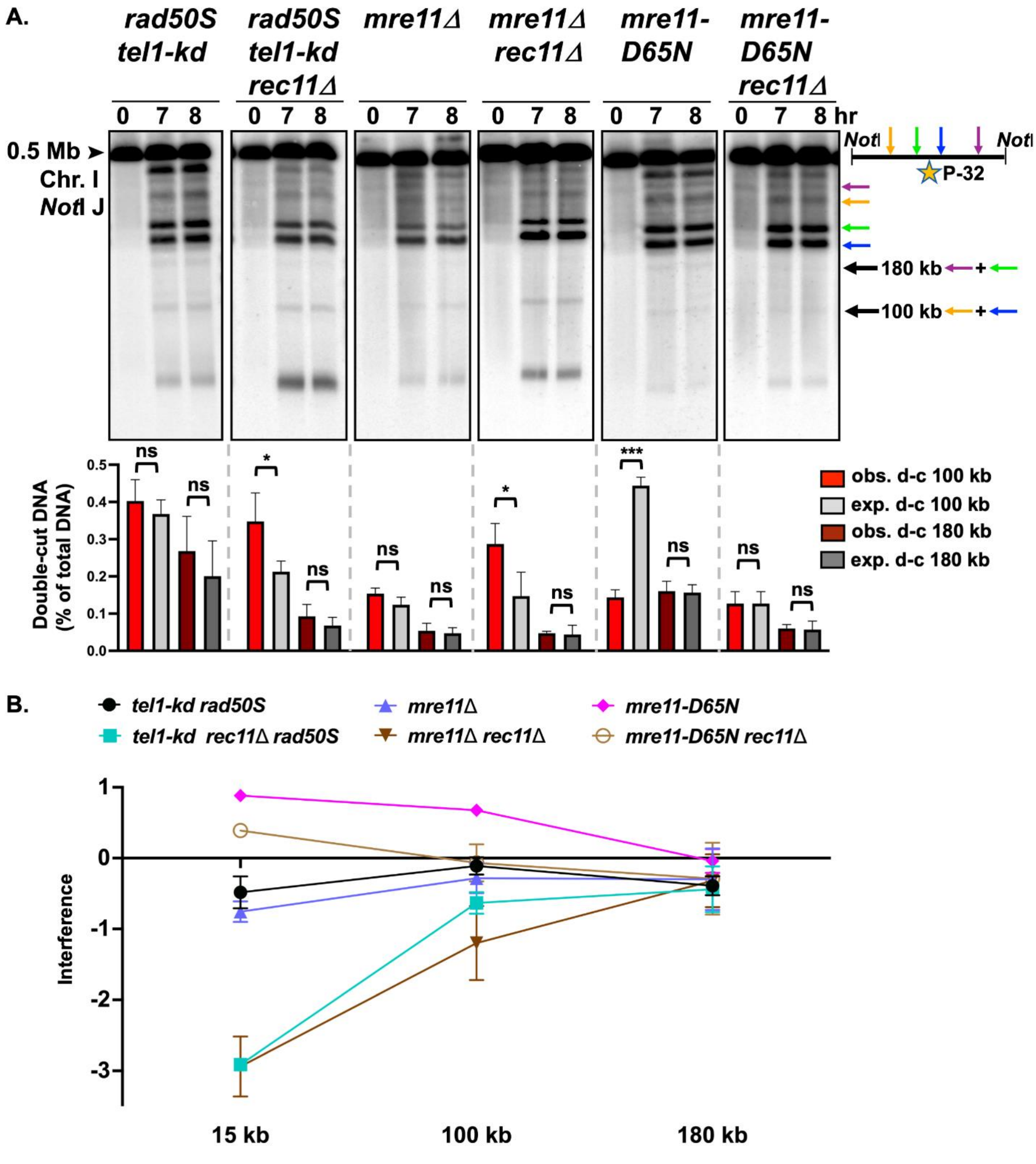

Figure S8. The strong negative DSB interference in *rec11*Δ is distance-dependent

A) The frequency of double-cut DNA between two additional hotspot pairs was measured in *tel1-kd*, *mre11*Δ, and *mre11-D65N*, with or without the cohesin subunit Rec11. *Not*I-digested DNA was Southern blot hybridized with a [^{32}P]-labelled DNA probe located in the middle of the 0.5 Mb *Not*I J fragment on Chromosome 1, between the inserted DSB hotspot *ura1::hph* and

mbs1 to detect DNA chromatids that were cut at both *mbs1* (blue arrow) and a DSB 100 kb away (*mbs2*, orange arrow), or at both *ura1::hph* (green arrow) and a DSB 180 kb away (purple arrow). The observed (red and maroon bars) and expected (% DSB 1 x % DSB 2; light and dark gray bars) double-cut frequencies (as % of total DNA) are plotted for both pairs of DSBs. Observed and expected values are the mean of the 7-and 8-hour timepoints. For each strain, the mean and SEM are shown from 3 or 4 experiments. (B) DSB interference, calculated as 1 – (observed double-cut DNA/expected double-cut DNA), for each strain is plotted on the graph, for DSB hotspot pairs at three distances – 15, 100, and 180 kb. Data for 15 kb (double-cut *mbs1* and *ura1::hph*) are from Figures 4 and 6. For each strain, the mean and SEM are shown from 3 or 4 experiment.

References

1. Hyppa, R.W., Cho, J.D., Nambiar, M. and Smith, G.R. (2022) Redirecting meiotic DNA break hotspot determinant proteins alters localized spatial control of DNA break formation and repair. *Nucleic Acids Res*, **50**, 899-914.
2. Guerra-Moreno, A., Alves-Rodrigues, I., Hidalgo, E. and Ayte, J. (2012) Chemical genetic induction of meiosis in *Schizosaccharomyces pombe*. *Cell Cycle*, **11**, 1621-1625.
3. Farah, J.A., Hartsuiker, E., Mizuno, K.-I., Ohta, K. and Smith, G.R. (2002) A 160-bp palindrome is a Rad50•Rad32-dependent mitotic recombination hotspot in *Schizosacchromyces. pombe*. *Genetics*, **161**, 461-468.
4. Grimm, C., Bahler, J. and Kohli, J. (1994) *M26* recombinational hotspot and physical conversion tract analysis in the *ade6* gene of *Schizosaccharomyces pombe*. *Genetics*, **135**, 41-51.
5. Fowler, K.R., Hyppa, R.W., Cromie, G.A. and Smith, G.R. (2018) Physical basis for long-distance communication along meiotic chromosomes. *Proc Natl Acad Sci U S A*, **115**, E9333-E9342.
6. Pryce, D.W., Lorenz, A., Smirnova, J.B., Loidl, J. and McFarlane, R.J. (2005) Differential activation of M26-containing meiotic recombination hot spots in *Schizosaccharomyces pombe*. *Genetics*, **170**, 95-106.
7. Kitajima, T.S., Yokobayashi, S., Yamamoto, M. and Watanabe, Y. (2003) Distinct cohesin complexes organize meiotic chromosome domains. *Science*, **300**, 1152-1155.
8. Martin-Castellanos, C., Blanco, M., Rozalen, A.E., Perez-Hidalgo, L., Garcia, A.I., Conde, F., Mata, J., Ellermeier, C., Davis, L., San-Segundo, P. *et al.* (2005) A large-scale screen in *S. pombe* identifies seven novel genes required for critical meiotic events. *Current Biology*, **22**, 2056-2062.
9. Davis, L. and Smith, G.R. (2003) Non-random homolog segregation at meiosis I in *Schizosaccharomyces pombe* mutants lacking recombination. *Genetics*, **163**, 857-874.
10. Estreicher, A., Lorenz, A. and Loidl, J. (2012) Mug20, a novel protein associated with linear elements in fission yeast meiosis. *Current Genetics*, **58**, 119-127.
11. Watanabe, Y. and Nurse, P. (1999) Cohesin Rec8 is required for reductional chromosome segregation at meiosis. *Nature*, **400**, 461-464.
12. Ueno, M., Nakazaki, T., Akamatsu, Y., Watanabe, K., Tomita, K., Lindsay, H.D., Shinagawa, H. and Iwasaki, H. (2003) Molecular characterization of the *Schizosaccharomyces pombe* *nbs1*$^{+}$ gene involved in DNA repair and telomere maintenance. *Molecular Cell Biology*, **23**, 6553-6563.
13. Wilson, S., Tavassoli, M. and Watts, F.Z. (1998) *Schizosaccharomyces pombe* rad32 protein: a phosphoprotein with an essential phosphoesterase motif required for repair of DNA double strand breaks. *Nucleic Acids Res*, **26**, 5261-5269.
14. Limbo, O., Chahwan, C., Yamada, Y., de Bruin, R.A., Wittenberg, C. and Russell, P. (2007) Ctp1 is a cell-cycle-regulated protein that functions with Mre11 complex to control double-strand break repair by homologous recombination. *Mol. Cell*, **28**, 134-146.
15. Manolis, K.G., Nimmo, E.R., Hartsuiker, E., Carr, A.M., Jeggo, P.A. and Allshire, R.C. (2001) Novel functional requirements for non-homologous DNA end joining in *Schizosaccharomyces pombe*. *EMBO Journal*, **20**, 210-221.
16. Latypov, V., Rothenberg, M., Lorenz, A., Octobre, G., Csutak, O., Lehmann, E., Loidl, J. and Kohli, J. (2010) Roles of Hop1 and Mek1 in meiotic chromosome pairing and recombination partner choice in *Schizosaccharomyces pomb*e. *Mol Cell Biol*, **30**, 1570-1581.
